# Transcriptional Responses of Cultured Rat Sympathetic Neurons during BMP-7-Induced Dendritic Growth

**DOI:** 10.1371/journal.pone.0021754

**Published:** 2011-07-13

**Authors:** Michelle M. Garred, Michael M. Wang, Xin Guo, Christina A. Harrington, Pamela J. Lein

**Affiliations:** 1 Gene Microarray Shared Resource, Oregon Health & Science University, Portland, Oregon, United States of America; 2 Departments of Neurology and Molecular & Integrative Physiology, University of Michigan, VA Ann Arbor Healthcare System, Ann Arbor, Michigan, United States of America; 3 Department of Environmental Health Sciences, Johns Hopkins University, Bloomberg School of Public Health, Baltimore, Maryland, United States of America; 4 Department of Molecular Biosciences, School of Veterinary Medicine, University of California Davis, Davis, California, United States of America; University of Turin, Italy

## Abstract

**Background:**

Dendrites are the primary site of synapse formation in the vertebrate nervous system; however, relatively little is known about the molecular mechanisms that regulate the initial formation of primary dendrites. Embryonic rat sympathetic neurons cultured under defined conditions extend a single functional axon, but fail to form dendrites. Addition of bone morphogenetic proteins (BMPs) triggers these neurons to extend multiple dendrites without altering axonal growth or cell survival. We used this culture system to examine differential gene expression patterns in naïve vs. BMP-treated sympathetic neurons in order to identify candidate genes involved in regulation of primary dendritogenesis.

**Methodology/Principal Findings:**

To determine the critical transcriptional window during BMP-induced dendritic growth, morphometric analysis of microtubule-associated protein (MAP-2)-immunopositive processes was used to quantify dendritic growth in cultures exposed to the transcription inhibitor actinomycin-D added at varying times after addition of BMP-7. BMP-7-induced dendritic growth was blocked when transcription was inhibited within the first 24 hr after adding exogenous BMP-7. Thus, total RNA was isolated from sympathetic neurons exposed to three different experimental conditions: (1) no BMP-7 treatment; (2) treatment with BMP-7 for 6 hr; and (3) treatment with BMP-7 for 24 hr. Affymetrix oligonucleotide microarrays were used to identify differential gene expression under these three culture conditions. BMP-7 significantly regulated 56 unique genes at 6 hr and 185 unique genes at 24 hr. Bioinformatic analyses implicate both established and novel genes and signaling pathways in primary dendritogenesis.

**Conclusions/Significance:**

This study provides a unique dataset that will be useful in generating testable hypotheses regarding transcriptional control of the initial stages of dendritic growth. Since BMPs selectively promote dendritic growth in central neurons as well, these findings may be generally applicable to dendritic growth in other neuronal cell types.

## Introduction

The shape of the dendritic arbor determines the total synaptic input a neuron can receive [1], [2], [3], and influences the types and distribution of these inputs [4], [5], [6]. Altered patterns of dendritic growth and plasticity are associated with impaired neurobehavioral function in experimental models [7], and are thought to contribute to clinical symptoms observed in both neurodevelopmental disorders [8], [9], [10] and neurodegenerative diseases [11], [12], [13]. Such observations underscore the functional importance of precisely regulating dendritic morphology and suggest that identifying mechanisms that control dendritic growth will not only advance understanding of how neuronal connectivity is regulated during normal development, but may also provide insight on novel therapeutic strategies for diverse neurological diseases.

Dendritic development can be broadly separated into two phases: primary dendrite formation, which includes initiation of dendritic growth and extension of primary dendritic shafts; and dendritic maturation, which encompasses dendrite branching and elongation, spine formation and dendritic retraction [14], [15]. While much research has focused on mechanisms that control dendritic maturation [16], [17], [18], [19], comparatively little is known about mechanisms that regulate primary dendritogenesis [14], [15]. It is generally thought that transcriptional mechanisms are required for the formation of primary dendrites [15], and genetic studies in *Drosophila* [15], [20], [21] have identified a number of transcription factors that are important in this initial phase of dendritic development in this model organism. Less is known, however, about gene expression patterns that control primary dendritogenesis in mammalian neurons [15].

Primary culture of dissociated sympathetic neurons offers a unique opportunity for addressing this gap in knowledge [22]. When cultured in the absence of serum and ganglionic glial cells, sympathetic neurons extend a single functional axon, but fail to form dendrites [23], [24]. However, addition of recombinant bone morphogenetic proteins (BMPs) triggers these neurons to extend multiple dendrites without altering axonal growth or cell survival [25], [26]. The dendritic arbor induced by BMPs in cultured sympathetic neurons is comparable to that of their *in vivo* counterparts with respect to size, accumulation and post-translational modification of dendrite-specific cytoskeletal and membrane proteins, exclusion of axonal proteins, transport of select mRNA, and formation of synaptic contacts of the appropriate polarity [25], [27], [28]. These observations indicate that BMPs selectively induce the execution of a developmental program in sympathetic neurons that controls both the quantitative and qualitative aspects of dendritic growth. Mechanistic studies of BMP-induced dendritic growth in this model system are consistent with the proposed role of transcriptional mechanisms in initial dendrite formation: 1) the canonical BMP signaling pathway involves activation of Smad transcription factors and Smad activation is required for BMP-induced dendritic growth [29]; and 2) pharmacological inhibition of transcription blocks the dendrite promoting activity of BMPs [28]. Therefore, to identify candidate genes involved in regulating primary dendritogenesis, we characterized global gene expression profiles in sympathetic neurons undergoing BMP-induced primary dendritogenesis using microarray analysis.

## Results

### BMP-7 triggers dendritogenesis in cultured sympathetic neurons via transcriptional mechanisms

**Figure 1:**
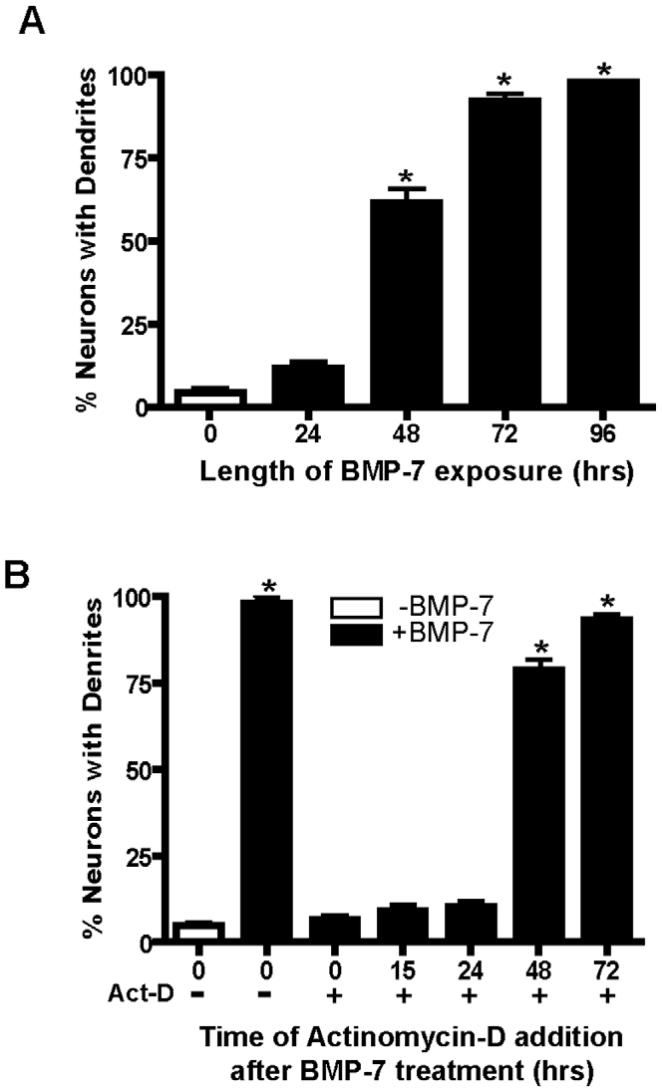
BMP-7-induced dendritic growth in cultured sympathetic neurons requires transcription. Sympathetic neurons dissociated from embryonic rat superior cervical ganglia (SCG) and cultured in defined medium in the absence of serum and non-neuronal cells were treated with BMP-7 (50 ng/ml) added to the medium on day 5 *in vitro*. (A) Cultures were fixed at varying times after BMP-7 addition and immunostained for MAP-2 to visualize dendritic processes. Significant dendritogenesis was evident within 48 hr after BMP-7 addition, and by 96 hr, over 95% of the neurons had responded to the dendrite-promoting activity of BMP-7. (B) Actinomycin-D (100 ng/ml) was added to sympathetic cultures at varying times after addition of BMP-7 (50 ng/ml). Following a 72 hr exposure to BMPs, dendritic growth was quantified in MAP-2 immunoreactive cells. Actinomycin-D inhibited dendritogenesis when added within 24 hr after BMP-7 treatment. BMP-7-induced dendritic growth was not blocked when actinomycin-D was added at 48 or 72 hr after BMP-7 treatment. Data presented as the mean ± S.E. (n = 3). *Significantly different from control (cultures grown in the absence of BMP-7) at *p*<0.001 (One-way ANOVA with *post-hoc* Tukey's analysis).

As previously reported [25], sympathetic neurons cultured in the absence of serum and ganglionic glial cells failed to extend dendrites; however, addition of BMP-7 to these cultures triggered a robust dendritic response (Figure 1). The extension of dendrites was not an immediate response to BMP-7, but rather became apparent at the structural level approximately 24–48 hr after BMP-7 was added to the culture medium (Figure 1A). The observation that less than 5% of neurons extended dendrites in the absence of exogenous BMP-7 while dendrites were elaborated by more than 95% of neurons exposed to BMP-7 demonstrated the high “signal-to-noise” ratio in this experimental model system.

To determine the critical period when transcriptional changes required for the dendritic response to BMP-7 occurred, transcription was inhibited by adding actinomycin-D to cultures at varying times after BMP-7 addition. When added within the first 24 hr after BMP-7 exposure, actinomycin-D effectively blocked BMP-induced dendritic growth; however, when added at 48 or 72 hr after BMP treatment, actinomycin-D had no significant effect on the dendrite promoting activity of BMP-7 (Figure 1B). Consistent with previous studies [27], the addition of BMP-7 in the absence or presence of actinomycin-D did not influence axonal growth as determined by western blotting of cell lysates from these cultures using antibodies specific for phosphorylated M and H neurofilament subunits, nor did these treatments influence cell survival as determined by propidium iodide uptake (data not shown). Based on these observations, total RNA for microarray analyses was harvested from primary cultures of sympathetic neurons at 6 hr and 24 hr after addition of BMP-7 to identify early and late transcriptional responses to BMP-7.

### Transcriptome analysis of dendritogenesis

Affymetrix Rat Genome U34A oligonucleotide microarrays were used to interrogate transcripts differentially regulated by BMP-7 in sympathetic neurons. Total RNA was collected from cultures derived from the same dissection and grown under identical conditions up until day 5 *in vitro*. At that time, a subset of cultures was exposed to BMP-7, which was added to the medium for varying periods of time resulting in 3 experimental conditions: (1) no BMP-7 treatment; (2) treatment with BMP-7 for 6 hr; and (3) treatment with BMP-7 for 24 hr. This experiment was independently repeated 3 times using cultures derived from 3 independent dissections, resulting in a total of 9 arrays (3 arrays for each of the 3 experimental conditions).

**Figure 2:**
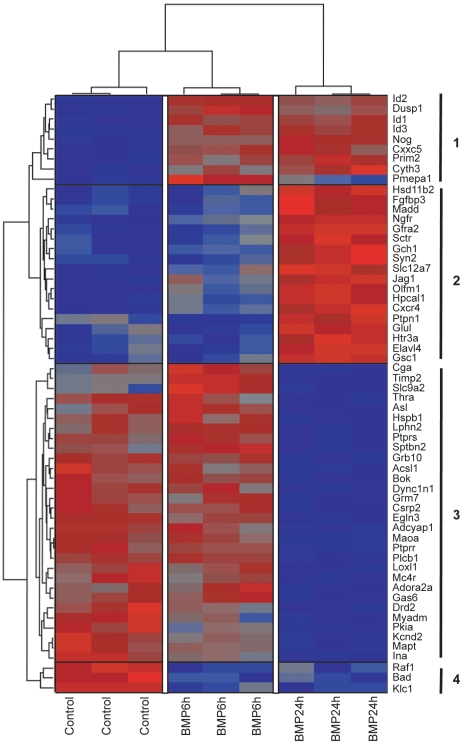
Cluster diagram of transcripts differentially regulated by BMP-7 in cultured sympathetic neurons. The 60 most significantly regulated transcripts (ANOVA, *p*<0.005) were analyzed by Partek Genomics Suite 6.5 to determine hierarchical clustering. Samples (columns) are clustered based on treatment condition whereas transcripts (rows) are clustered based on expression pattern. Relative levels of gene expression are depicted with a color scale in which red represents the highest level of up-regulated expression and blue represents the lowest level of down-regulated expression. Unsupervised clustering identified 4 major groups of genes, identified by the solid lines and numbers on the far right. Group 1 represents genes that are upregulated by 6 hr after BMP-7 treatment and remain upregulated at 24 hr after BMP-7 treatment; Group 2, genes upregulated only at 24 hr post-BMP-7 addition; Group 3, genes that are downregulated by BMP-7 at 24 hr; and Group 4, genes that are downregulated by 6 hr after BMP-7 treatment and remain downregulated at 24 hr after BMP-7 treatment. Additional information for the genes identified in Groups 1 and 2 is provided in Table S1; genes identified in Groups 3 and 4 are described in more detail in Table S2.

The data set was analyzed by two-way ANOVA to identify a set of genes regulated by BMP-7 (the complete data set is available from the GEO repository, accession number GSE28150). Significant changes (*p*<0.005) across the BMP-7 treatment course were identified, and patterns of gene expression changes over the sample set were visualized using hierarchical clustering (Figure 2, Tables S1 and S2). The hierarchical clustering revealed 4 basic gene expression changes: (1) genes upregulated by BMP-7 at 6 hr that remained upregulated at 24 hr; (2) genes upregulated by BMP-7 only at 24 hr; (3) genes downregulated by BMP-7 at 24 hr; and (4) genes downregulated by BMP-7 at 6 hr that remained downregulated at 24 hr. The name and *p*-value of each gene identified in this hierarchical clustering are listed in Tables S1 and S2 for genes up- and down-regulated by BMP-7, respectively.

**Figure 3:**
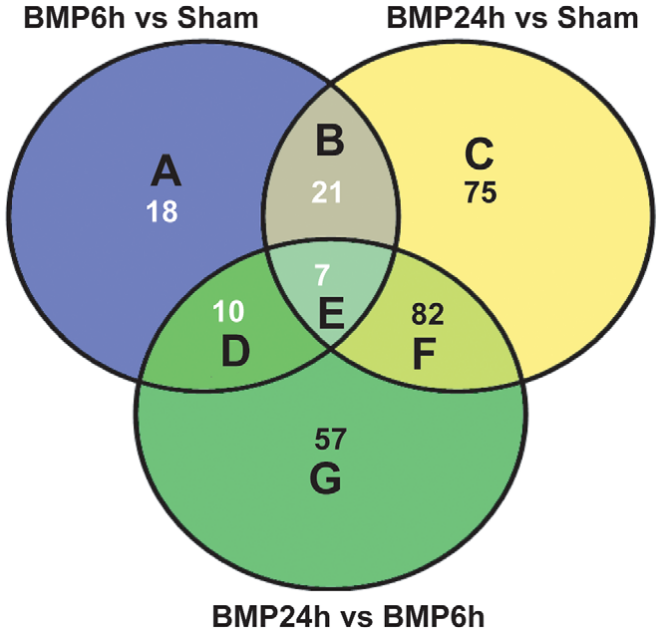
Overview of gene expression changes after addition of BMP-7 to sympathetic neurons. Venn diagram showing concordance of significant changes in transcript levels between treatment comparisons. The numbers in each section refer to the number of annotated genes that were found to be significantly different between treatment groups (*p*<0.05, 1.2 fold change up or down). Specific genes corresponding to groups identified by the letters A through G are identified in Tables S3, S4, S5 available in on-line supporting information.

An analysis of significant differences between treatment conditions identified a total of 270 genes as significantly changed by BMP-7 treatment (*p*<0.05; 1.2 fold change filter). As illustrated in a Venn diagram (Figure 3), 56 annotated genes were differentially regulated following 6 hr of BMP-7 treatment relative to control; 185 annotated genes were differentially regulated following 24 hr of BMP-7 treatment relative to control; and 156 annotated genes were differentially regulated following 24 hr versus 6 hr of BMP-7 treatment (see Tables S3, S4, S5 for more information on the genes included in these categories). More genes are regulated at 24 hr than at 6 hr after BMP-7 addition, with 50% (28 genes) of the genes regulated at 6 hr also found in the regulated gene set at 24 hr.

**Figure 4:**
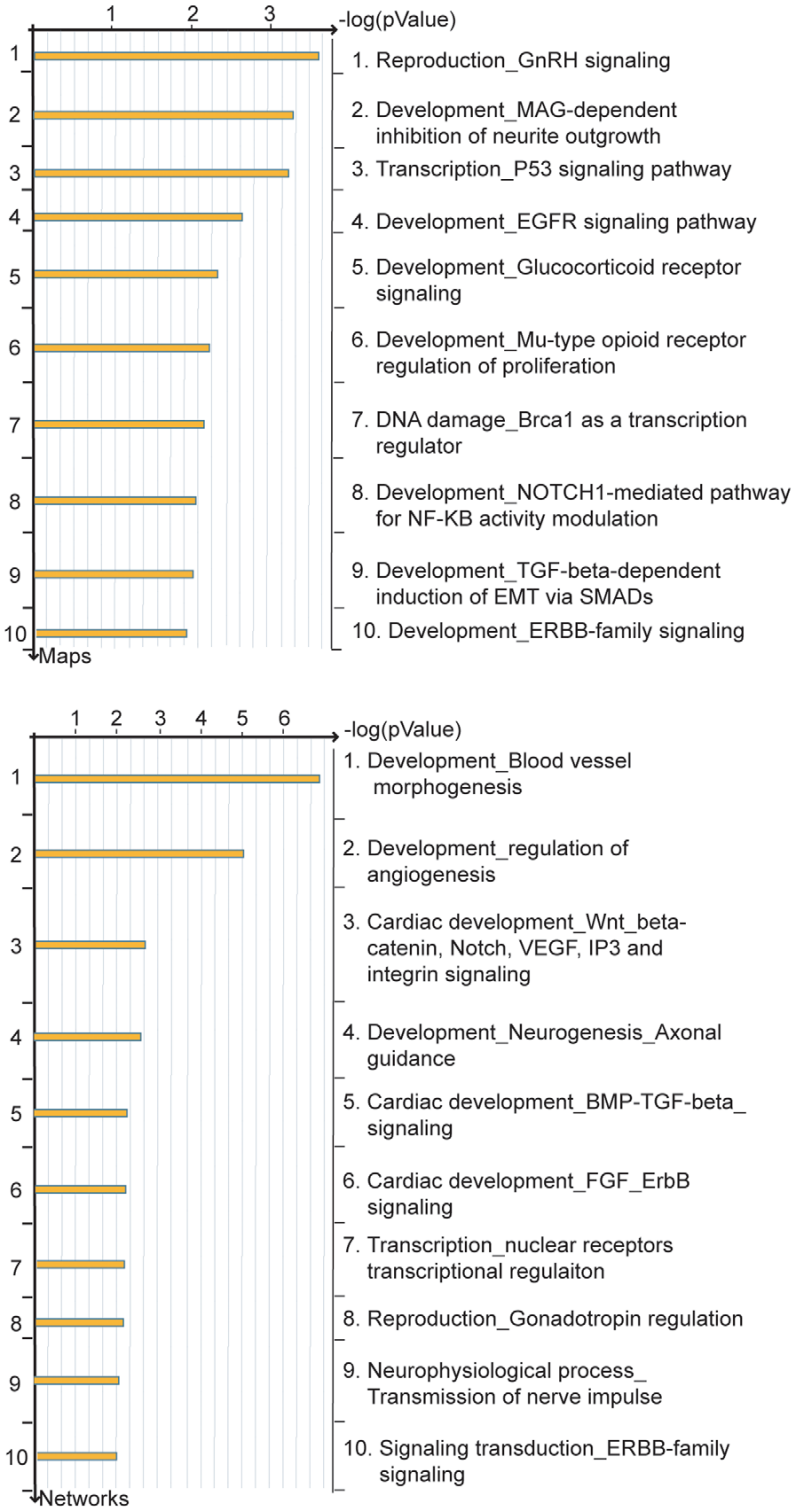
Functional analysis of genes differentially regulated during dendritogenesis in cultured sympathetic neurons. Probe sets that were determined to be significantly regulated in sympathetic neurons exposed to BMP-7 for 6 hr (compared to controls) were analyzed using MetaCore software (GeneGo). The most significant Gene Ontology signaling and metabolic (top panel) and cellular and molecular (bottom panel) pathways are shown.

Pathway analysis of genes differentially regulated by BMP-7 at 6 hr identified several canonical signaling pathways that could be activated by BMP-7 (Figure 4). This analysis demonstrated that primary dendritogenesis in cultured sympathetic neurons is accompanied by changes in gene expression that parallel the regulatory pathways and signaling networks that guide general development and branching morphogenesis. The signaling pathway with the lowest *p*-value was a pathway important in reproduction, the gonadotropin releasing hormone (GnRH) signaling pathway (*p* value 2.589e-4). In addition, several signaling pathways previously implicated in neuronal morphogenesis were identified as showing significant relationships with genes differentially regulated by BMP-7 in sympathetic neurons during primary dendritogenesis. These included the myelin associated glycoprotein (MAG)-dependent inhibition of neurite outgrowth signaling pathway (*p* value 5.427e-4), the NOTCH-1 mediated pathway for NF-κB activity modulation (*p* value 9.599e-3) and the Smad-dependent transforming growth factor (TGF)-β signaling pathway (*p* value 1.015e-2). Of the GeneGo Process Networks, the top five statistically significant results were (1) development, blood vessel morphogenesis (*p* value 1.470e-07), (2) development, regulation of angiogenesis (*p* value 1.020e-05), (3) cardiac development, Wnt, beta-catenin, Notch, VEGF, IP3, and integrin signaling (*p* value 2.70e-3), (4) development, neurogenesis, axonal guidance (*p* value 3.441e-3), and (5) cardiac development, BMP, TGF beta signaling (*p* value 6.727e-03).

**Figure 5:**
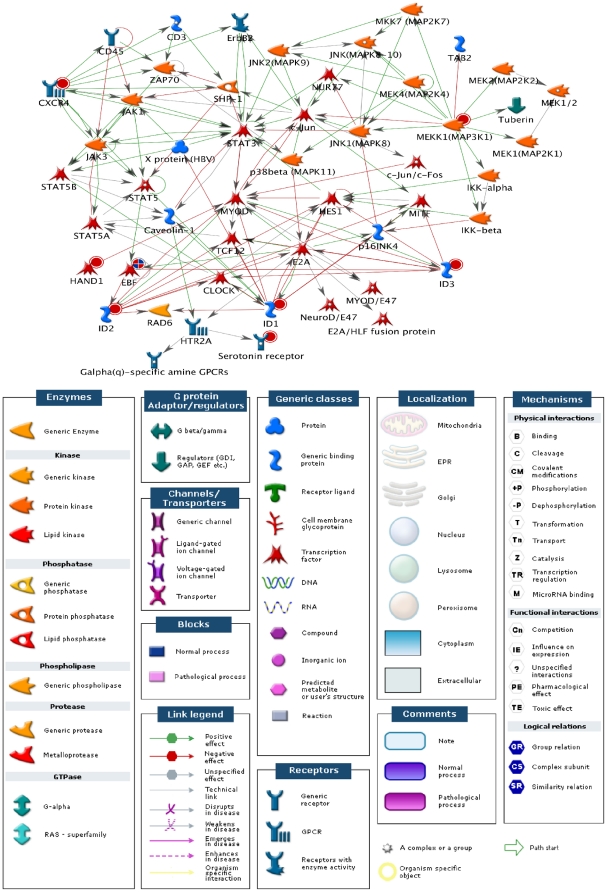
Informatic analysis of interactions between BMP-7-regulated genes identifies potential roles of Id transcriptional repressors. Transcripts differentially regulated by BMP-7 at 6 hr (*p*<0.05, 1.2 fold change up or down; listed in Tables S3, S4, S5) were entered into the GenGO Analyze Networks (AN) algorithm with default settings. Shown is one of the top scored networks as identified by an enrichment z-score. Thick cyan lines indicate the fragments of canonical pathways. Up-regulated genes from the gene input list are marked with red circles. A significant network of pathways linked by annotated functional data included three of the *Id* genes, which are among the most robust transcriptional regulators identified in our analysis and which are found in the same cluster of genes activated at both 6 and 24 hr after BMP-7 treatment.

To identify potential interactions between biological networks, the profile of transcripts differentially regulated by BMP-7 at 6 hr was analyzed using the GeneGO Analyze Networks algorithm. Several gene interaction webs were identified, but the one that included the most transcripts identified as regulated by BMP-7 included the inhibitor of DNA binding (Id) family of transcription repressors (Figure 5). The *Id* genes were upregulated strongly at both 6 and 24 hr (Figure 2). Interestingly, the *Id* genes identified in these microarray analyses are closely linked to canonical signaling pathways important in development, and many of them are implicated in direct or indirect control of expression of other genes identified as differentially regulated by BMP-7 in sympathetic neurons, including *Hand1* [30], [31], *Ebf* [32], [33], [34], *Ngfr* (also known as *p75*) [35] and *Cxcr4* [36], [37]. The acute upregulation of these *Id* transcriptional regulators and their control of other genes in different expression clusters suggest a role in immediate early control of BMP-7 responses.

### Experimental validation of BMP-induced gene expression

**Figure 6:**
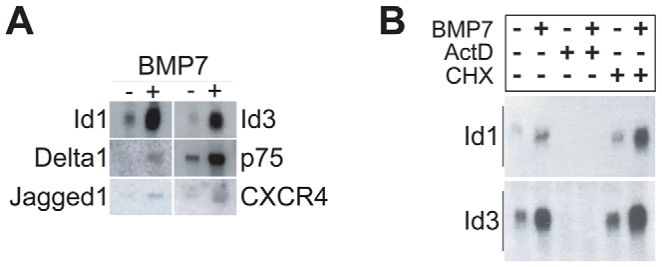
Validation of BMP-7 induction of expression for genes identified as up-regulated in microarray analysis. To validate results of microarray analysis of gene expression, we analyzed gene expression in primary cultures of sympathetic neurons treated with or without BMP-7 for 24 hr. Total RNA collected from these cultures was submitted to Northern blot analysis using six independent probes corresponding to genes identified as upregulated by microarray analysis. (**A**) Representative Northern blots probed for Id1, Id3, Delta1, Ngfr (p75), Jagged1 and CXCR4. In all cases, a single band was detected at the expected size, and equal loading of mRNA was verified by probing with actin, which was considered a housekeeping gene. (**B**) Effect of transcriptional and translational inhibitors on BMP-7-stimulated gene expression in sympathetic neurons. Cultures treated with or without BMP-7 in the absence or presence of actinomycin-D or cycloheximide to inhibit mRNA or protein synthesis, respectively. Total RNA from these cultures was analyzed by Northern blotting for expression of Id1 and Id3. BMP-7 induced expression of both Id1 and Id3 was blocked by actinomycin-D, while cycloheximide enhanced the expression of both genes in response to BMP-7.

To validate microarray results, Northern blot analysis was used to compare transcript levels of target genes in control cultures of sympathetic neurons *versus* sister cultures derived from the same dissection treated with BMP-7 for 24 hr. Target genes included a subset of genes listed in Table S1: *Id1*, *Id3*, *Ngfr* (also known as *p75*), *Cxcr4* and *Jag1*. In addition, we assessed BMP-7 effects on transcript levels of *Delta 1* (*Dll1*), which was not identified as a statistically significant BMP-7 regulated gene in the microarray data set but demonstrated a trend to upregulation and was of biological interest. Using independent probes, we confirmed that BMP-7 upregulated each of these target genes (Figure 6A). Transcript levels for two of the most robustly upregulated genes in BMP-7 treated sympathetic neurons, *Id1* and *Id3*, were significantly decreased by actinomycin-D but not cyclohexamide (Figure 6B), demonstrating that the BMP-7 upregulation of these genes required active transcription and was not affected by inhibition of protein translation.

## Discussion

Dendritic morphogenesis is a critical determinant of neuronal connectivity in the developing nervous system and an essential component of functional plasticity of the nervous system throughout life. A critical phase in dendritic morphogenesis is the initial formation of primary dendrites, yet little is known about the molecular changes that drive primary dendritogenesis. In this study we demonstrate that transcription is required for primary dendritogenesis in primary cultures of sympathetic neurons since pharmacologic inhibition of transcription by actinomycin-D inhibits BMP-7-induced dendritogenesis when administered during the first 24 hr after BMP-7 exposure. Notably, the number of genes differentially regulated by BMP-7 at 24 hr is significantly increased compared to the number of genes regulated at 6 hr, which is consistent with a defined genetic program that drives primary dendritogenesis. Our findings also represent, to our knowledge, the first unbiased transcriptome analysis of mammalian neurons during primary dendritogenesis. Genes identified as differentially regulated by BMP-7 in cultured sympathetic neurons form a discreet dataset of candidate genes involved in primary dendritogenesis that will be useful in directing novel hypothesis-driven research into the molecular mechanisms that control the initial stages of dendrite formation. Below, we discuss the internal validity and relevance of this dataset, some of the unexpected expression patterns observed in this study, and potential strategies for using these data in mechanistic studies of dendritogenesis.

### Internal validation and relevance of these microarray data

The validity of the microarray data generated in this study is supported by two lines of evidence. First, Northern blot analysis confirmed microarray data for a subset of genes identified as upregulated by BMP-7. Second, multiple genes identified in this study as differentially regulated by BMP-7 have been reported to be similarly regulated by BMPs in other model systems. For example, *Adcyap* (PACAP) has been shown to be negatively regulated by BMPs [38] and was also suppressed in BMP-7-treated sympathetic neurons. Conversely, *Hand1* [39], [40], the *Id* genes [41], *Noggin* [42], [43], [44], *Ngfr* [45], *Klf10* [46], and *Vegf* [47] are upregulated by BMPs in other model systems and by BMP-7 in cultured sympathetic neurons. Comparable patterns of gene regulation across these studies not only validate our microarray data but also suggest the existence of a subset of conserved, cell-type independent BMP-responsive genes that constitute a canonical BMP signaling pathway across multiple BMP family members. However, other genes shown to be upregulated by BMPs in other model systems, notably, *Igfbp3* [48], [49], *Ccl2* [50] and *Cdh2* [51], were downregulated by BMP-7 in sympathetic neurons. This suggests that our dataset also includes genes that are uniquely regulated in our novel neuronal model system.

Although the identification of core BMP-regulated genes provides validity to our findings, these data also point to a potential limitation of our study, which is that at least a subset of genes regulated in our model could be BMP-responsive but not play a direct role in dendrite formation. However, several characteristics of the model system strongly suggest that the transcriptome described in this study is enriched for genes of functional relevance to primary dendritogenesis. One important characteristic is that our model is comprised of a homogenous population of sympathetic neurons devoid of other cell types [22], [25]. In addition, we were able to experimentally isolate synchronized neurons at two distinct stages of dendritic growth: immediately preceding the formation of dendrites and during the initial formation of primary dendrites. Third, neurons in these cultures respond uniformly to the dendrite-promoting activity of BMP-7 and other BMPs of the dpp and 60A subfamilies (Figure 1 and [25]), and BMP-induced dendrite formation occurs in the absence of changes in cell survival or axonal growth (data not shown and [25]). These characteristics significantly increased the likelihood of capturing genes specifically involved in dendrite formation rather than genes that regulate general neurite formation, neuronal differentiation or neuronal cell survival. This conclusion is corroborated by comparisons between the microarray dataset generated in this study and transcriptomes reported from differentiating PC12 cells, which do not elaborate dendrites. Genes identified in this study are likely specific for dendrite (and not neurite) outgrowth since the set of genes differentially regulated by BMP-7 in cultured sympathetic neurons included only a few genes previously associated with NGF-induced neurite outgrowth in PC12 cells (*Calb1* [52], *Cited2* [53], *Egr1* [54], *Hspb1* [55], *Ptprr* [56] and *Tyro3* [57]) or identified as differentially regulated by a close family member of BMP-7, BMP-4, in PC12 cells (*Mapt* and *Egr1* [58]).

### Unexpected findings

Surprisingly, few genes that encode the major cytoskeletal proteins found in dendrites [59] were identified as transcriptionally regulated by BMP-7 during primary dendritogenesis in sympathetic neurons. One possibility is that since cytoskeletal proteins are downstream effectors, they are upregulated between the 24 and 48 hours-post-BMP treatment, which are the time points between which we observed a substantial difference in actinomycin treatment on BMP-7-induced dendritic growth. An alternative possibility is that in addition to transcriptional regulation, post-transcriptional mechanisms that regulate cytoskeleton proteins are critically important in dendrite formation. For example, tubulin production has been shown to involve translational feedback regulation that results in tight regulation of protein based on intracellular concentrations of unpolymerized subunits [60]). Additional transcriptome analyses at time points between 24 and 48 hours post-BMP treatment integrated with proteomic analysis may offer unique insights into additional effector genes and the role of non-transcriptional regulatory mechanisms that contribute to primary dendritogenesis.

Another unexpected finding was that multiple genes previously reported to activate growth of new neurites in primary neuronal cell cultures or neuronal cell lines were repressed by BMP-7 in sympathetic neurons, including: *Alcam* [61], *Areg* [62], *Atf3* [63], *Calb1* [64], [65], *Ccl2* [66], *Cd47* [67], [68], [69], *Cdh2* [69] and *Dclk1* [68]. Although the reasons for this discrepancy are unclear, there are several possible explanations. One technical consideration is that some of the initial experiments defining these genes as activators of neurite extension used overexpression paradigms, which may not reflect physiological function. Another possibility is that stimulation of the genetic program for primary dendritogenesis activates a negative feedback loop that functions to limit dendritic growth. Indeed the BMP-7 transcriptome in sympathetic neurons suggests a role for both positive and negative feedback regulation. One of the few genes downregulated by BMP-7 in sympathetic neurons was *Mgp*, which has been shown to inhibit the effects of BMPs in muscle [70]. The attenuation of *Mgp* would be predicted to accentuate BMP signaling and feed forward to sensitize cells to BMP-7. On the other hand, several genes activated by BMP-7 in cultured sympathetic neurons have previously been shown to antagonize BMP-mediated signals, including *Noggin*, and *Tmeff1* [71]. The transcription factor *Atf3*, which is a permissive protein that facilitates the activation of Id by BMPs [72], is downregulated by BMP-7 treatment of SCG neurons. The regulation of multiple genes that inhibit effects of BMP suggests the existence of negative feedback systems that are activated within 24 hr of BMP-7 exposure. Presumably, these negative feedback pathways are activated to prevent interminable signaling and thus prevent overgrowth of dendrites.

Other possible explanations for why genes shown to activate neurite outgrowth in other systems were downregulated by BMP-7 in sympathetic neurons include: 1) these genes have opposite effects on dendrites versus axons; and 2) the genetic program that drives BMP-7-induced dendritic growth in sympathetic neurons differs from genetic control of dendritic growth triggered by other stimuli in other neuronal cell types. With respect to the former, only a minority of previously published studies of neurite outgrowth identified the affected neurites as dendrites, so it is likely that the neurites stimulated by upregulation of these genes were axonal in nature. There is significant experimental evidence that axons and dendrites are differentially regulated [22], even by the same signaling molecule [73]. Regarding the latter, it is possible that these gene products have different roles in the context of BMP-induced dendritic growth in sympathetic neurons relative to their roles in other model systems of dendritic growth. There is precedence for this possibility: activation of the GTPase RhoA is required for BMP-7-induced dendritic growth in sympathetic neurons [74], but inhibits activity-dependent dendritic growth in cultured neurons of central origin [75], [76]. It is not clear whether this reflects a difference between peripheral or central neurons or between BMP-induced versus activity-dependent dendritic growth. It should be possible to distinguish between these possibilities experimentally since BMPs have been shown to robustly enhance dendritic growth in central neurons in culture [77], [78], [79], [80].

### Potential strategies for using these data in mechanistic studies of dendritogenesis

Gene transfection, pharmacological modulation, and siRNA can be used in cultured sympathetic neurons to directly assess the functional role of BMP-regulated genes in primary dendritogenesis and several studies are underway in our lab to investigate individual genes using these approaches. However, given the large number of BMP-regulated genes discovered in our analysis, gene-by-gene functional evaluation of physiological regulators of dendritogenesis is challenging. One approach that might be useful in prioritizing genes for mechanistic studies is to focus on transcripts whose gene products are localized to dendrites, which implies a direct role in dendritogenesis, rather than upregulation as an epiphenomenon. Such genes include *Cxcr4* [81], [82], *Hpcal1* [83], [84], *Gfra2* [85], *Ina* [86], *Pka* [87], [88], *Syt4* [89], [90], and *Tyro3* [91].

Another approach for prioritizing genes for gene-by-gene analysis is to identify genes that have already been shown to participate in dendrite or, less stringently, neurite outgrowth. Indeed, several of the BMP-7-responsive genes activated in sympathetic neurons during dendritogenesis have been previously shown to modulate dendritic growth in other model systems. Examples include *Jagged1*, a ligand for the Notch system that has been implicated in controlling multiple aspects of dendritic growth [75], [92], [93]; *Mark1*, a gene whose optimal levels of expression is required for proper dendrite length [94]; *Ngfr* (*p75*), which has complex roles in dendrite stabilization and likely modulates increased sensitivity to dendrite regulating ligands [95], [96], [97]; *Pka*, which responds to increased cAMP levels that play a major role in dendrite formation and stability [88], [98], [99], [100] and *Vegfa*, which plays a role in dendrite formation during development [101], [102] and numerous pathophysiological states [103]. Conversely, we found downregulation of several genes that have been demonstrated to inhibit dendrite or neurite formation, including *Adcyap1* (PACAP) [104], *Maoa* and *Maob* [105], [106], [107], [108] and *Pdlim1* whose expression in sensory neurons downregulates neurite number [109]. The combined upregulation of a set of pro-dendritic genes and downregulation of anti-dendritic genes may underlie the robust response of sympathetic neurons to BMP-7. It remains to be determined whether these genes play a role in primary dendritogenesis versus dendrite modulation, but these genes are certainly potentially important based on functions established in independent studies and seem likely to be functionally relevant in primary dendritogenesis in sympathetic neurons.

A final strategy is to investigate functional relationships using pathway analyses. Figure 5 highlights an example of a complex network of known interactions inferred using the set of genes differentially regulated by BMP-7 at 6 hr. The analysis suggests that *Id* genes play an important role in the regulation of a number of genes that are differentially regulated by BMP-7 in sympathetic neurons, which in turn suggests that *Id* genes play a central and important early role in regulating primary dendritogenesis. Ids are known transcriptional regulators that control expression of downstream genes, and our experimental data (Figure 6) confirm that *Id* genes are strongly upregulated by BMP-7 and meet the criteria for immediate early genes in that they respond rapidly to exogenous factors but do not require new protein translation. As has been observed with other immediate early gene responses, cycloheximide enhanced the relative abundance of Id1 and Id3 mRNAs in the absence of BMP treatment. This phenomenon may be due to impairment of inhibitory factors that require protein synthesis (e.g., basal transcription of Id1 could be inhibited by a transcription factor that is rapidly turning over and protein synthesis inhibitors therefore deplete this transcription factor resulting in lower Id1 levels).

### Conclusion

In conclusion, we present the first comprehensive gene profiling analysis of a model system for primary dendritogenesis in a mammalian neuron. Our findings identify a set of genes differentially regulated by BMP-7 in sympathetic neurons during primary dendritogenesis. Bioinformatic analyses implicate a number of well-established and novel genes and signaling pathways, which will inform testable hypotheses regarding transcriptional control of the initial stages of dendritic growth. That these findings may be generally applicable to dendritic growth in other neuronal cell types is suggested by evidence that BMPs also selectively promote dendritic growth in central neurons [77], [78], [79], [80], [110].

## Materials and Methods

### Ethics Statement

All procedures involving animals were performed according to protocol number RA02H321 approved by the Institutional Animal Care and Use Committee at Johns Hopkins University. Timed-pregnant Holtzman rats were purchased from Harlan (Indianapolis, IN) and housed individually in standard plastic cages with Alpha-Dri bedding (Shepherd Specialty Papers, Watertown, TN) in a temperature (22±2°C) controlled room on a 12 h reverse light-dark cycle. Food and water were provided *ad libitum*. Dams and pups were humanely euthanized prior to harvesting of tissues for culture; no experimental manipulations were performed prior to euthanasia.

### Primary Culture of Sympathetic Neurons

Sympathetic neurons were dissociated from the superior cervical ganglia (SCG) of embryonic day 21 (E21) rat pups and maintained in the absence of glial cells in serum-free medium supplemented with nerve growth factor (β-NGF, 100 ng/ml, Harlan Bioproducts, Indianapolis, IN) as previously described [111]. Recombinant human BMP-7 (50 ng/ml), which was a generous gift of Creative Biomolecules (now known as Curis, Cambridge, MA) was added to the medium on day 5 *in vitro* to trigger dendritic growth [25].

### Morphological analyses

To visualize dendrites, cultures were immunostained with a monoclonal antibody to MAP-2 (Sternberger-Meyer Immunochemicals, Jarrettsville, MD) and antigen∶antibody complexes were detected by indirect immunofluorescence as previously described (Lein *et al.*, 1995). In all morphometric analyses, only isolated neurons, i.e., neurons whose cell were bodies were at least 100 µm from the soma of the nearest neighboring cell were analyzed, since a previous study [23] demonstrated that density-dependent changes in cellular morphology occur when cell bodies are separated by lesser distances. Processes were scored as dendrites if they were MAP-2-immunoreactive, tapered over their length and at least equal in length to the diameter of the cell body. The percentage of neurons with dendrites was determined from 5 independent fields (at 200× magnification) per culture in 3 cultures per condition. These values were averaged within treatment groups to obtain the percentage of neurons with dendrites per treatment for that experiment. The experiment was repeated 3 times using cultures derived from 3 independent dissections, resulting in an n = 3 for statistical analysis of treatment-related effects using a one-way ANOVA with *p*<0.05 considered significant, followed by *post hoc* comparison of means using Student Newman-Keuls analysis.

### Western blot analysis of cytoskeletal proteins

To assess the effects of BMP treatment on axonal growth, cultured sympathetic neurons were plated onto poly-d-lysine coated 35 mm dishes and a subset of these cultures were treated with 50 ng/ml of BMP-7 in the presence or absence of actinomycin D for 24 hr beginning on day 5 *in vitro*. Cell lysates were collected from both control and BMP-7-treated cultures on day 6 *in vitro* by scraping cells off dishes in 50 mM Tris buffer (pH 7.4) containing 0.1% sodium dodecyl sulfate, 2% 2-mecaptoethanol and 1 mM EDTA and homogenized by passaging through a 23 gauge needle at 4°C. Cell lysates were centrifuged at 12,000× g for 15 min and the protein concentrations of the supernatants were determined using the Bradford dye reagent (Bio-Rad). Equal amounts of proteins were resolved by SDS-PAGE, electrophoretically transferred onto a nitrocellulose membrane, and probed with antibodies to MAP2 or an antibody to the phosphorylated forms of the H and M neurofilament subunits (SMI31; Sternberger Monoclonals), which are primarily found in axons. Immunoreactive bands were detected using Chemiluminescent Substrate (Pierce Chemical) after sequential treatment with biotinylated goat anti-mouse IgG (HyClone) and with horseradish peroxidase-conjugated streptavidin (Amersham).

### RNA Isolation and Microarray Processing

Total RNA was isolated from cultures grown under 3 different experimental conditions: 1) cultures not treated with BMP-7, referred to as control; 2) cultures exposed to BMP-7 for 6 hr, referred to as BMP6h; and 3) cultures exposed to BMP-7 for 24 hr, referred to as BMP24h. Total RNA was isolated from each pooled sample using the Qiagen RNeasy kit (Qiagen, Valencia, CA) per the manufacturer's protocol. In our experience, 5 µg total RNA is typically purified from one million neurons. The quality and concentration of the isolated RNA was assessed by spectrophotometry (2100 Bioanalyzer, Agilent Technologies, Santa Clara, CA) and gel electrophoresis. One RNA sample (pooled from multiple cultures set up at the same cell densities used for morphological analyses) of each experimental condition was collected per experiment from cultures derived from the same dissection and experiments were repeated 3 times using cultures derived from 3 independent samples resulting in nine total samples (3×3 study design). Total RNA was isolated from all experimental conditions at the same time (day 6 *in vitro*).

Gene expression microarray assays were performed in the Affymetrix Microarray Core of Johns Hopkins University Bloomberg School of Public Health (Baltimore, MD) following the 3′IVT one-cycle labeling and amplification protocol described in the Affymetrix GeneChip Expression Analysis Technical Manual (http://www.affymetrix.com/support/technical/manual/expression_manual_affx). Samples were processed in two batches: the first batch included 3 samples (with one biological replicate sample of 10 µg cRNA per each of the three experimental conditions) whereas the second batch included 6 samples (with two biological replicate samples of 5 µg cRNA per each of the three experimental conditions). Experimental conditions were balanced across the two batches in order to prevent a technical processing bias.

Samples were randomized prior to processing. Target material (cRNA) resulting from the labeling and amplification reactions was hybridized to Affymetrix GeneChip® Rat Genome U34A arrays, which contain oligonucleotide probe sets for 8,799 rat genes from full length mRNA transcripts and EST clusters. Target cRNA was hybridized over two hyb/scan processing batches; the quantity of cRNA hybridized varied by batch (10 µg cRNA hybridized for Batch1 and 5 µg cRNA hybridized for Batch2). Arrays were scanned using MAS5 software (Affymetrix, Santa Clara, CA) to produce raw image data (DAT files) and raw probe cell level signal intensity values (CEL files). Analysis of array performance quality metrics was completed by the Gene Microarray Shared Resource of Oregon Health & Science University (Portland, OR) using Affymetrix GeneChip Command Console software (Affymetrix, Santa Clara, CA) and custom scripts for data visualization.

### Microarray data analysis

Affymetrix CEL files were imported to Partek Genomics Suite v. 6.5 (Partek, St. Louis, MO) for data visualization and statistical testing. Upon data upload, pre-processing of CEL data for the complete data set (total of nine samples; three biological replicate samples each for Control, BMP6h, and BMP24h conditions) was performed using the Robust MultiChip Average (RMA) algorithm [112], [113]. A two-way ANOVA statistical test was performed, including factors for treatment (control, BMP6h, BMP24h) and processing batch (Batch1: control, BMP6h, BMP24h; Batch 2: two samples each of control, BMP6h, BMP24h); the Partek software “batch removal” function was evoked for the latter factor, which performs a signal value adjustment aimed to minimize batch-specific technical variation. The Partek batch removal function is designed for cases when batching is balanced across the sample conditions (User's Guide, Partek Genomics Suite). Differential gene expression across the treatment course was assessed by applying a filter on *p*-value (Treatment)<0.005 to the ANOVA results (note that all reported *p*-values are unadjusted and therefore not corrected for multiple testing). Patterns of gene expression from this analysis output were visualized using hierarchical clustering. For a more in depth analysis of the between treatment differences, three linear contrasts were performed: BMP6h versus control, BMP24h versus control, and BMP24h versus BMP6h. Lists of significant genes were generated for each comparison. Gene list criteria included passing a maximum 0.05 raw *p*-value cutoff and a minimum fold-change (FC) cut-off of 1.2 for both up- and down-regulated genes in response to BMP treatment. Concordance of the gene lists was visualized by Venn diagram (http://bioinfogp.cnb.csic.es/tools/venny/index.html). Gene function and pathway annotation were associated with the data set using MetaCore software (GeneGo, St. Joseph, MI).

All microarray data is MIAME compliant and the raw data has been deposited in the MIAME compliant GEO database (accession number GSE28150).

### Northern blot analysis

Sympathetic neuronal cell cultures were rinsed with phosphate buffered saline immediately prior to RNA purification, and then treated with Trizol (Invitrogen, Carlsbad, CA) to solubilize nucleic acids. The Trizol suspension was extracted as recommended by the manufacturer and RNA was precipitated with isopropanol and rinsed with 70% ethanol prior to resuspension in water. RNA (5–10 µg) was treated with formamide (Sigma, St. Louis, MO) and heated to 65°C prior to electrophoresis on formaldehyde agarose gels. RNA gels were blotted using conventional capillary transfer onto Whatman Nytran™ membranes (GE Healthcare, Piscataway, NJ). Membranes were pre-blocked in hybridization buffer (Stratagene QuickHyb, Agilent Technologies, La Jolla, CA) prior to exposure to denatured, random-primed ^32^P-labeled probes. After overnight exposure, the filters were washed at high stringency in 0.1% SDS and 0.1× SSC (saline-sodium citrate; diluted from 20× SSC containing 3 M sodium chloride and 300 mM sodium citrate, pH 7.0) at 65°C, followed by autoradiography for 2 hr to 2 wk.

Probes used for hybridization experiments were derived by excising mouse clones from plasmid vectors. After restriction digestion of plasmids, fragments were gel purified and then random prime labeled using the Stratagene Prime-IT kit, Agilent Technologies) as previously described [114]. Unless specified, the probes included the complete open reading frame and variable portions of untranslated sequences. Id probes were fully sequenced cDNA clones generated by Dr. Greg Kato (National Institutes of Health, Bethesda, MD). Under hybridization and washing conditions used in these experiments, there was no cross reaction between the Id isoforms on Northern blotting. The *Ngfr* (*p75*) probe was a 1.0 kb XbaI/BamHI fragment released from p288, a clone encoding the complete open reading frame of *Ngfr* (a generous gift from Phillip Barker, Montreal Neurological Institute, McGill University, Montreal Canada), which represents the 3′ half of the coding sequence plus part of the 3′ non-coding region. Macaque CXCR4 was isolated from the plasmid pCR-Rh-CSCR4.2, which was obtained from the NIH AIDS Research & Reference Reagent Program (Rockville, MD). Probes for human Jagged1 and Delta1 were excised from full-length clones (gifts from Nicholas Gaiano, Johns Hopkins University School of Medicine, Baltimore, MD).

## Supporting Information

Table S1 Genes upregulated by BMP-7 in cultured sympathetic neurons.
(DOC)

Table S2 Genes downregulated by BMP-7 in cultured sympathetic neurons.
(DOC)

Table S3 Genes significantly changed at BMP6h relative to control (56 total). ^a^Venn diagram illustrated in Figure 3 of artcle; ^b^FC = fold change.
(DOC)

Table S4 Genes significantly changed at BMP24h relative to control (185 total). ^a^Venn diagram illustrated in Figure 3; ^b^FC = fold change.
(DOC)

Table S5 Genes significantly changed at BMP24h relative to BMP6h (156 total). ^a^Venn diagram illustrated in Figure 3; ^b^FC = fold change.
(DOC)
