# Supplementary material for: Transcriptional Responses of Cultured Rat Sympathetic Neurons during BMP-7-Induced Dendritic Growth
Source: PLoS One. 2011 Jul 13;6(7):e21754. doi: 10.1371/journal.pone.0021754 (PMC3135585; doi:10.1371/journal.pone.0021754)
Supplement: Table S1 — Genes upregulated by BMP-7 in cultured sympathetic neurons. (DOC) [file pone.0021754.s001.doc]

**Table S1. Genes upregulated by BMP-7 in cultured sympathetic neurons**

| **Cluster #** | **Gene Symbol** | **p-value (Treatment)** | **Probeset ID** | **Gene Title** | **RefSeq Transcript ID** |
| --- | --- | --- | --- | --- | --- |
| **1** | Cxxc5 | 0.00062 | rc_H33001_at | CXXC finger 5 | NM_001007628 |
| **1** | Cyth3 | 0.00445 | U83897_at | cytohesin 3 | NM_053912 |
| **1** | Id1 | 0.00234 | L23148_g_at | inhibitor of DNA binding 1 | NM_012797 |
| **1** | Id2 | 0.00007 | rc_AI137583_at | inhibitor of DNA binding 2 | NM_013060 |
| **1** | Id3 | 0.00055 | AF000942_at | inhibitor of DNA binding 3 | NM_013058 |
| **1** | Nog | 0.00054 | U31203_at | noggin | NM_012990 |
| **1** | Pmepa1 | 0.00123 | rc_AI639058_s_at | prostate transmembrane protein, androgen induced | NM_001107807 |
| **1** | Prim2 | 0.0004 | AJ011607_at | primase, DNA, polypeptide | NM_001024762 |
| **2** | Cxcr4 | 0.00068 | U90610_at | chemokine (C-X-C motif) receptor 4 | NM_022205 |
| **2** | Elavl4 | 0.00244 | S83320_g_at | ELAV (embryonic lethal, abnormal vision)-like 4 (Hu antigen D) | NM_001077651 |
| **2** | Fgfbp3 | 0.00485 | rc_AA800782_at | fibroblast growth factor binding protein 3 | NM_001109165 |
| **2** | Gch1 | 0.00178 | M58364_at | GTP cyclohydrolase 1 | NM_024356 |
| **2** | Gcs1 | 0.00418 | AF087431_g_at | glucosidase 1 | NM_031749 |
| **2** | Gfra2 | 0.00014 | U97143_at | GDNF family receptor alpha | NM_012750 |
| **2** | Glul | 0.00451 | M91652complete_seq_g_at | glutamate-ammonia ligase (glutamine synthetase) | NM_017073 |
| **2** | Hpcal1 | 0.00041 | D13126_at | hippocalcin-like 1 | NM_017356 |
| **2** | Hsd11b2 | 0.00153 | U22424_at | hydroxysteroid 11-beta dehydrogenase 2 | NM_017081 |
| **2** | Htr3a | 0.00233 | U59672_at | 5-hydroxytryptamine receptor 3a | NM_024394 |
| **2** | Jag1 | 0.00261 | L38483_at | jagged 1 | NM_019147 |
| **2** | Madd | 0.00262 | U72995_at | MAP-kinase activating death domain | NM_053585 |
| **2** | Ngfr | 0.00035 | X05137_at | nerve growth factor receptor *aka* p75 (TNFR superfamily, member 16) | NM_012610 |
| **2** | Olfm1 | 0.00013 | U03414_s_at | olfactomedin 1 | NM_053573 |
| **2** | Ptpn1 | 0.00199 | M33962_g_at | protein tyrosine phosphatase, non-receptor | NM_012637 |
| **2** | Sctr | 0.0029 | E04128cds_s_at | secretin receptor | NM_031115 |
| **2** | Slc12a7 | 0.00235 | rc_AA799691_at | solute carrier family 12 (potassium/chloride transporters), member 7 | XM_001060536 /// XM_001071999 |
| **2** | Syn2 | 0.00413 | M27925_at | synapsin II | NM_001034020 /// NM_019159 |
|  |  |  |  |  |  |
