# Supplementary material for: Transcriptional Responses of Cultured Rat Sympathetic Neurons during BMP-7-Induced Dendritic Growth
Source: PLoS One. 2011 Jul 13;6(7):e21754. doi: 10.1371/journal.pone.0021754 (PMC3135585; doi:10.1371/journal.pone.0021754)
Supplement: Table S2 — Genes downregulated by BMP-7 in cultured sympathetic neurons. (DOC) [file pone.0021754.s002.doc]

**Table S**2. Genes downregulated by BMP-7 in cultured sympathetic neurons

| **Cluster #** | **Gene Symbol** | **p-value (Treatment)** | **Probeset ID** | **Gene Title** | **RefSeq Transcript ID** |
| --- | --- | --- | --- | --- | --- |
| **3** | Acsl1 | 0.0041 | D90109_at | acyl-CoA synthetase long-chain family member 1 | NM_012820 |
| **3** | Adcyap1 | 0.00436 | X80290cds_s_at | adenylate cyclase activating polypeptide 1 | NM_016989 |
| **3** | Adora2a | 0.00454 | S47609_s_at | adenosine A2a receptor | NM_053294 |
| **3** | Asl | 0.00183 | AB016536_s_at | argininosuccinate lyase | NM_021577 |
| **3** | Bok | 0.00134 | AF027954_at | BCL2-related ovarian killer | NM_017312 |
| **3** | Cga | 0.00181 | AF016702mRNA_at | glycoprotein hormones, alpha polypeptide | NM_053918 |
| **3** | Csrp2 | 0.0002 | U44948_at | cysteine and glycine-rich protein 2 | NM_177425 |
| **3** | Drd2 | 0.00362 | X56065_s_at | dopamine receptor D2 | NM_012547 |
| **3** | Dusp1 | 0.00018 | S81478_s_at | dual specificity phosphatase 1 | NM_053769 |
| **3** | Dync1h1 | 0.00165 | L08505_at | dynein cytoplasmic 1 heavy chain 1 | NM_019226 |
| **3** | Egln3 | 0.00007 | rc_AA799678_s_at | EGL nine homolog 3 | NM_019371 |
| **3** | Gas6 | 0.00073 | D42148_at | growth arrest specific 6 | NM_057100 |
| **3** | Grb10 | 0.00018 | rc_AA800686_at | growth factor receptor bound protein 10 | NM_001109093 |
| **3** | Grm7 | 0.00202 | D16817_at | glutamate receptor, metabotropic 7 | NM_031040 |
| **3** | Hspb1 | 0.00262 | rc_AA998683_g_at | heat shock protein 1 | NM_031970 |
| **3** | Ina | 0.00082 | rc_AA875659_s_at | Internexin,intermediate filament alpha | NM_019128 |
| **3** | Kcnd2 | 0.00325 | M59980_s_at | potassium voltage-gated channel, Shal-related subfamily, member 2 | NM_031730 |
| **3** | Loxl1 | 0.00235 | rc_AA859805_at | lysyl oxidase-like 1 | NM_001012125 |
| **3** | Lphn2 | 0.00025 | AF063102_at | latrophilin 2 | NM_134408 |
| **3** | Maoa | 0.00228 | S45812_s_at | monoamine oxidase A | NM_033653 |
| **3** | Mapt | 0.00345 | X79321_at | microtubule-associated protein tau | NM_017212 |
| **3** | Mc4r | 0.00353 | U67863_at | melanocortin 4 receptor | NM_013099 |
| **3** | Myadm | 0.00315 | rc_AA866276_at | myeloid-associated differentiation marker | NM_183332 |
| **3** | Pkia | 0.0033 | rc_AA893743_g_at | Protein kinase inhibitor alpha | NM_053772 |
| **3** | Plcb1 | 0.00064 | L14323_at | phospholipase C, beta 1 | NM_001077641 |
| **3** | Ptprr | 0.00204 | D64050_at | tyrosine phosphatase, receptor type, R | NM_001113390 /// NM_053594 |
| **3** | Ptprs | 0.00063 | L19933_s_at | tyrosine phosphatase, receptor type, S | NM_019140 |
| **3** | Slc9a2 | 0.00135 | L11236UTR#1_s_at | solute carrier family 9 (sodium/hydrogen exchanger), member 2 | NM_001113335 /// NM_012653 |
| **3** | Sptbn2 | 0.00055 | AB001347_s_at | spectrin, beta, non-erythrocytic 2 | NM_019167 |
| **3** | Thra | 0.00356 | M31174_at | thyroid hormone receptor alpha | NM_001017960 /// NM_031134 |
| **3** | Timp2 | 0.00014 | S72594_s_at | TIMP metallopeptidase inhibitor 2 | NM_021989 |
| **4** | Bad | 0.00464 | rc_AA818072_s_at | BCL2-associated agonist of cell death | NM_022698 |
| **4** | Klc1 | 0.00117 | M75148_at | kinesin light chain 1 | NM_001081972 /// NM_001081973 /// NM_001081974 |
| **4** | Raf1 | 0.00216 | M15428 | v-raf-leukemia viral oncogene 1 | NM_012639 |
