# Supplementary material for: Transcriptional Responses of Cultured Rat Sympathetic Neurons during BMP-7-Induced Dendritic Growth
Source: PLoS One. 2011 Jul 13;6(7):e21754. doi: 10.1371/journal.pone.0021754 (PMC3135585; doi:10.1371/journal.pone.0021754)
Supplement: Table S3 — Genes significantly changed at BMP6h relative to control (56 total). aVenn diagram illustrated in Figure 3 of artcle; bFC = fold change. (DOC) [file pone.0021754.s003.doc]

**Table S3. Genes significantly changed at BMP6h relative to control (56 total).**

| **Venn Diagram Section^a^** | **Gene Symbol** | **FC^b^ BMP6h vs Control** | **FC BMP24h vs Control** | **FC BMP24h vs BMP6h** | | **Probe Set ID** | **GO Biological Process** |
| --- | --- | --- | --- | --- | --- | --- | --- |
| **A** | Efna1 | 1.64 | ns | ns | rc_AA892417_at | | activation of MAPK activity |
| **A** | Man2c1 | -1.26 | ns | ns | rc_AA946384_at | | carbohydrate metabolic process |
| **A** | Cdh22 | 1.73 | ns | ns | D83348_at | | cell adhesion |
| **A** | Mlf1 | -1.29 | ns | ns | rc_AA800632_at | | cell cycle arrest |
| **A** | Hal | -1.35 | ns | ns | AB002393_at | | histidine metabolic process |
| **A** | Il12b | 1.21 | ns | ns | U16674_at | | natural killer cell activation during immune response |
| **A** | Pdp1 | 1.61 | ns | ns | AF062740_at | | protein amino acid dephosphorylation |
| **A** | Pctk2 | 1.46 | ns | ns | AB005540_at | | protein amino acid phosphorylation |
| **A** | Fxc1 | -1.2 | ns | ns | rc_AA891857_g_at | | protein transport |
| **A** | Ebf1 | -1.28 | ns | ns | L24051_at | | regulation of transcription, DNA-dependent |
| **A** | Tjp1 | -1.35 | ns | ns | rc_AA892918_at | | response to lipopolysaccharide |
| **A** | Vom2r31 | -1.34 | ns | ns | AF016184_at | | signal transduction |
| **A** | P2ry2 | 1.73 | ns | ns | U56839_at | | signal transduction |
| **A** | Col1a1 | 1.24 | ns | ns | M27207mRNA_s_at | | skeletal system development |
| **A** | Acadvl | -1.21 | ns | ns | D30647_at | | temperature homeostasis |
| **A** | Rpl3l | -1.31 | ns | ns | rc_AA891037_g_at | | translation |
| **A** | Eef2 | 1.21 | ns | ns | rc_AA963674_at | | translational elongation |
| **A** | Junb | 1.48 | ns | ns | rc_AA891041_at | | vasculogenesis |
| **B** | Vegfa | 1.25 | 1.23 | ns | L20913_s_at | | angiogenesis |
| **B** | Hand1 | 1.78 | 1.66 | ns | Y08140_at | | angiogenesis |
| **B** | Unc5b | 1.3 | 1.37 | ns | U87306_at | | apoptosis |
| **B** | Atf3 | -1.57 | -2.02 | ns | M63282_at | | cell fate determination |
| **B** | Cdo1 | -1.55 | -2.25 | ns | rc_AA942685_at | | cysteine metabolic process |
| **B** | Klf6 | -1.38 | -1.23 | ns | AF001417_s_at | | cytokine-mediated signaling pathway |
| **B** | Hbegf | 1.98 | 1.66 | ns | L05489_at | | epidermal growth factor receptor signaling pathway |
| **B** | Areg | -1.89 | -2.47 | ns | X55183_at | | epidermal growth factor receptor signaling pathway |
| **B** | Gadd45a | -1.47 | -1.45 | ns | rc_AI070295_g_at | | G2/M transition of mitotic cell cycle |
| **B** | Klf10 | 1.64 | 1.67 | ns | rc_AI172476_at | | induction of apoptosis |
| **B** | Id1 | 3.98 | 4.1 | ns | L23148_g_at | | negative regulation of transcription from RNA polymerase II promoter |
| **B** | Cited2 | -1.69 | -1.73 | ns | rc_AA900476_g_at | | negative regulation of transcription from RNA polymerase II promoter |
| **B** | Id3 | 3.39 | 3.26 | ns | rc_AI171268_at | | negative regulation of transcription from RNA polymerase II promoter |
| **B** | Nog | 4.29 | 7.14 | ns | U31203_at | | osteoblast differentiation |
| **B** | Cxxc5 | 1.65 | 1.72 | ns | rc_H33001_at | | positive regulation of I-kappaB kinase/NF-kappaB cascade |
| **B** | Mcpt3 | -1.37 | -1.35 | ns | U67888_at | | proteolysis |
| **B** | Ppat | 1.78 | 2.17 | ns | D10853_at | | purine nucleotide biosynthetic process |
| **B** | Cyth3 | 1.25 | 1.4 | ns | U83897_at | | regulation of ARF protein signal transduction |
| **B** | Gabbr2 | 1.21 | 1.23 | ns | AF058795_at | | signal transduction |
| **B** | Htr1d | 1.42 | 1.4 | ns | M89953cds_at | | signal transduction |
| **B** | Pelo | -1.23 | -1.24 | ns | rc_AA799330_at | | translation |
| **D** | Hsd17b6 | -1.3 | ns | 1.35 | U89280_at | | androgen metabolic process |
| **D** | Nr3c1 | -1.36 | ns | 1.35 | rc_AA893618_s_at | | chromatin remodeling |
| **D** | Klf9 | 1.87 | ns | -1.71 | D12769_g_at | | embryo implantation |
| **D** | Map3k1 | 1.56 | ns | -1.59 | U48596_g_at | | MAPKKK cascade |
| **D** | Prrxl1 | 2 | ns | -2.01 | U29174_at | | neuron migration |
| **D** | Rxrg | 1.7 | ns | -1.5 | AF016387_g_at | | regulation of transcription, DNA-dependent |
| **D** | Imp3 | -1.4 | ns | 1.3 | rc_AA799369_at | | rRNA processing |
| **D** | Ttr | -1.32 | ns | 1.31 | rc_AA945169_at | | transport |
| **D** | Gse1 | 1.43 | ns | -1.24 | rc_AI013993_at | | n/a |
| **D** | Pmepa1 | 1.86 | ns | -1.55 | rc_AI639058_s_at | | n/a |
| **E** | Ngfr | 1.66 | 3.77 | 2.27 | X05137_at | | apoptosis |
| **E** | Jag1 | 1.66 | 2.84 | 1.71 | L38483_at | | cell fate determination |
| **E** | Olfm1 | 1.25 | 1.79 | 1.43 | U03414_s_at | | multicellular organismal development |
| **E** | Id2 | 5.37 | 3.96 | -1.36 | rc_AI137583_at | | negative regulation of transcription from RNA polymerase II promoter |
| **E** | Dusp1 | 2.21 | 1.78 | -1.24 | S81478_s_at | | protein amino acid dephosphorylation |
| **E** | Cxcr4 | 1.44 | 2.82 | 1.96 | U90610_at | | response to hypoxia |
| **E** | Hpcal1 | 1.36 | 3.15 | 2.31 | D13126_at | | n/a |

^a^Venn diagram illustrated in Figure 3; ^b^FC = fold change.
