# Supplementary material for: Transcriptional Responses of Cultured Rat Sympathetic Neurons during BMP-7-Induced Dendritic Growth
Source: PLoS One. 2011 Jul 13;6(7):e21754. doi: 10.1371/journal.pone.0021754 (PMC3135585; doi:10.1371/journal.pone.0021754)
Supplement: Table S4 — Genes significantly changed at BMP24h relative to control (185 total). aVenn diagram illustrated in Figure 3; bFC = fold change. (DOC) [file pone.0021754.s004.doc]

**Table S4. Genes significantly changed at BMP24h relative to control (185 total)**

| **Venn Diagram Section^a^** | **Gene Symbol** | **FC^b^ BMP6h vs Control** | **FC BMP24h vs Control** | **FC BMP24h vs BMP6h** | **Probe Set ID** | **GO Biological Process** |
| --- | --- | --- | --- | --- | --- | --- |
| **B** | Vegfa | 1.25 | 1.23 | ns | L20913_s_at | angiogenesis |
| **B** | Hand1 | 1.78 | 1.66 | ns | Y08140_at | angiogenesis |
| **B** | Unc5b | 1.3 | 1.37 | ns | U87306_at | apoptosis |
| **B** | Atf3 | -1.57 | -2.02 | ns | M63282_at | cell fate determination |
| **B** | Cdo1 | -1.55 | -2.25 | ns | rc_AA942685_at | cysteine metabolic process |
| **B** | Klf6 | -1.38 | -1.23 | ns | AF001417_s_at | cytokine-mediated signaling pathway |
| **B** | Hbegf | 1.98 | 1.66 | ns | L05489_at | epidermal growth factor receptor signaling pathway |
| **B** | Areg | -1.89 | -2.47 | ns | X55183_at | epidermal growth factor receptor signaling pathway |
| **B** | Gadd45a | -1.47 | -1.45 | ns | rc_AI070295_g_at | G2/M transition of mitotic cell cycle |
| **B** | Klf10 | 1.64 | 1.67 | ns | rc_AI172476_at | induction of apoptosis |
| **B** | Id1 | 3.98 | 4.1 | ns | L23148_g_at | negative regulation of transcription from RNA polymerase II promoter |
| **B** | Cited2 | -1.69 | -1.73 | ns | rc_AA900476_g_at | negative regulation of transcription from RNA polymerase II promoter |
| **B** | Id3 | 3.39 | 3.26 | ns | rc_AI171268_at | negative regulation of transcription from RNA polymerase II promoter |
| **B** | Nog | 4.29 | 7.14 | ns | U31203_at | osteoblast differentiation |
| **B** | Cxxc5 | 1.65 | 1.72 | ns | rc_H33001_at | positive regulation of I-kappaB kinase/NF-kappaB cascade |
| **B** | Mcpt3 | -1.37 | -1.35 | ns | U67888_at | proteolysis |
| **B** | Ppat | 1.78 | 2.17 | ns | D10853_at | purine nucleotide biosynthetic process |
| **B** | Cyth3 | 1.25 | 1.4 | ns | U83897_at | regulation of ARF protein signal transduction |
| **B** | Gabbr2 | 1.21 | 1.23 | ns | AF058795_at | signal transduction |
| **B** | Htr1d | 1.42 | 1.4 | ns | M89953cds_at | signal transduction |
| **B** | Pelo | -1.23 | -1.24 | ns | rc_AA799330_at | translation |
| **C** | Madd | ns | 1.21 | ns | U72995_at | activation of MAPK activity |
| **C** | Dgka | ns | 1.32 | ns | S49760_g_at | activation of protein kinase C activity by G-protein coupled receptor protein signaling pathway |
| **C** | Atp2c1 | ns | 1.43 | ns | M93017_at | ATP biosynthetic process |
| **C** | Chrna9 | ns | -1.23 | ns | U12336_at | cation transport |
| **C** | Alcam | ns | -1.78 | ns | AB008538_at | cell adhesion |
| **C** | Rom1 | ns | 1.31 | ns | rc_AA892855_at | cell adhesion |
| **C** | Ninj1 | ns | 1.22 | ns | U72660_at | cell adhesion |
| **C** | Cbx5 | ns | 1.43 | ns | rc_AA893788_at | chromatin assembly or disassembly |
| **C** | Nfib | ns | -1.39 | ns | AB012231_s_at | DNA replication |
| **C** | Slbp | ns | 1.29 | ns | rc_AI639172_s_at | DNA replication involved in S phase |
| **C** | Hip1 | ns | 1.85 | ns | rc_AA859948_at | endocytosis |
| **C** | Ykt6 | ns | 1.2 | ns | AF033027_at | ER to Golgi vesicle-mediated transport |
| **C** | Hacl1 | ns | 1.43 | ns | rc_AA893239_at | fatty acid alpha-oxidation |
| **C** | Cdh2 | ns | -1.82 | ns | AF097593_g_at | homophilic cell adhesion |
| **C** | Pdgfra | ns | 1.2 | ns | rc_AI137657_at | in utero embryonic development |
| **C** | Diablo | ns | -1.34 | ns | rc_AA891933_at | induction of apoptosis |
| **C** | Nell1 | ns | -1.21 | ns | U48246_at | induction of apoptosis |
| **C** | Cxcl1 | ns | -1.35 | ns | D11445exon#1-4_s_at | inflammatory response |
| **C** | Phgdh | ns | -1.25 | ns | rc_AI008677_s_at | L-serine biosynthetic process |
| **C** | Dync1h1 | ns | -1.2 | ns | L08505_at | microtubule-based movement |
| **C** | Dctn1 | ns | -1.27 | ns | X62160_at | microtubule-based process |
| **C** | Hnrnpa1 | ns | 1.23 | ns | rc_AA799893_g_at | mRNA processing |
| **C** | Cabp1 | ns | 1.29 | ns | Y17048_g_at | multicellular organismal development |
| **C** | Rarb | ns | -1.38 | ns | AJ002942cds_at | negative regulation of transcription from RNA polymerase II promoter |
| **C** | Zfp148 | ns | 1.86 | ns | U30381_at | negative regulation of transcription from RNA polymerase II promoter |
| **C** | Dclk1 | ns | -1.45 | ns | AF030089UTR#1_at | neuron migration |
| **C** | Cntn2 | ns | 1.25 | ns | M31725_at | neuron migration |
| **C** | Sv2b | ns | -2.33 | ns | L10362_at | neurotransmitter uptake |
| **C** | Ak1 | ns | 1.21 | ns | D13376_at | nucleobase, nucleoside, nucleotide and nucleic acid metabolic process |
| **C** | H2afz | ns | 1.2 | ns | M37584_at | nucleosome assembly |
| **C** | Slk | ns | 1.32 | ns | rc_H31623_s_at | nucleotide-excision repair |
| **C** | Mgp | ns | -1.39 | ns | rc_AI012030_at | ossification |
| **C** | Vgf | ns | -1.33 | ns | M74223_at | ovarian follicle development |
| **C** | P4hb | ns | 1.32 | ns | M21476_s_at | peptidyl-proline hydroxylation to 4-hydroxy-L-proline |
| **C** | Slc25a10 | ns | 1.31 | ns | AJ223355_g_at | phosphate transport |
| **C** | Nptn | ns | -1.21 | ns | X99338cds_i_at | positive regulation of protein amino acid phosphorylation |
| **C** | Ptpn5 | ns | 1.92 | ns | S49400_at | protein amino acid dephosphorylation |
| **C** | Limk1 | ns | -1.21 | ns | D31873_g_at | protein amino acid phosphorylation |
| **C** | Tyro3 | ns | -1.84 | ns | D37880_at | protein amino acid phosphorylation |
| **C** | Ntrk1 | ns | 1.21 | ns | M85214_at | protein amino acid phosphorylation |
| **C** | Npepo | ns | 1.47 | ns | rc_AA799396_g_at | proteolysis |
| **C** | Kcnd2 | ns | -1.24 | ns | M59980_s_at | regulation of action potential |
| **C** | Esrra | ns | 1.42 | ns | rc_AA799412_g_at | regulation of ossification |
| **C** | Snf8 | ns | 1.31 | ns | rc_AA891666_at | regulation of transcription from RNA polymerase II promoter |
| **C** | Nr2f6 | ns | 1.26 | ns | rc_AA801029_s_at | regulation of transcription, DNA-dependent |
| **C** | Myt1l | ns | 1.24 | ns | U67081_at | regulation of transcription, DNA-dependent |
| **C** | Sst | ns | 1.63 | ns | M25890_at | response to acid |
| **C** | Casp3 | ns | -1.55 | ns | U84410_s_at | response to hypoxia |
| **C** | Ccl2 | ns | -1.52 | ns | X17053cds_s_at | response to hypoxia |
| **C** | Anxa5 | ns | -1.21 | ns | D42137exon_s_at | response to organic substance |
| **C** | Cyss | ns | -1.24 | ns | J04206_s_at | response to protozoan |
| **C** | Furin | ns | 1.57 | ns | X55660_g_at | signal peptide processing |
| **C** | Gnb5 | ns | -1.25 | ns | AF001953_at | signal transduction |
| **C** | Lphn1 | ns | 1.26 | ns | AF081144_s_at | signal transduction |
| **C** | Sstr1 | ns | -1.65 | ns | M97656_s_at | signal transduction |
| **C** | Arhgap28 | ns | 1.71 | ns | rc_AA891588_at | signal transduction |
| **C** | Crisp2 | ns | -1.45 | ns | AB009662_at | spermatogenesis |
| **C** | Mrps11 | ns | 1.27 | ns | rc_AA859788_at | translation |
| **C** | Slc25a30 | ns | 1.49 | ns | rc_AA892522_at | transmembrane transport |
| **C** | Cyb5a | ns | -1.47 | ns | AF007107_s_at | transport |
| **C** | Syt4 | ns | -1.6 | ns | U14398_g_at | transport |
| **C** | Kcnh1 | ns | 1.57 | ns | Z34264_at | two-component signal transduction system (phosphorelay) |
| **C** | Atp2b1 | ns | -1.34 | ns | L04739cds_s_at | n/a |
| **C** | Crygf | ns | -1.2 | ns | M19357cds_f_at | n/a |
| **C** | Myadm | ns | -1.28 | ns | rc_AA866276_at | n/a |
| **C** | Man1a1 | ns | -1.69 | ns | rc_AA892549_at | n/a |
| **C** | Pkia | ns | -1.21 | ns | rc_AA893743_g_at | n/a |
| **C** | Nptx1 | ns | 2.03 | ns | rc_AI072943_at | n/a |
| **C** | Psat1 | ns | -1.39 | ns | rc_AI230228_at | n/a |
| **C** | Ssb | ns | 1.36 | ns | S59893_f_at | n/a |
| **C** | Camk2g | ns | -1.66 | ns | S71570_s_at | n/a |
| **C** | Scg3 | ns | -1.29 | ns | U02983_at | n/a |
| **C** | Wipf3 | ns | -1.3 | ns | U31160mRNA_s_at | n/a |
| **C** | Ifitm3 | ns | -1.51 | ns | X61381cds_s_at | n/a |
| **C** | Pnoc | ns | 1.32 | ns | X97374exon_g_at | n/a |
| **E** | Ngfr | 1.66 | 3.77 | 2.27 | X05137_at | apoptosis |
| **E** | Jag1 | 1.66 | 2.84 | 1.71 | L38483_at | cell fate determination |
| **E** | Olfm1 | 1.25 | 1.79 | 1.43 | U03414_s_at | multicellular organismal development |
| **E** | Id2 | 5.37 | 3.96 | -1.36 | rc_AI137583_at | negative regulation of transcription from RNA polymerase II promoter |
| **E** | Dusp1 | 2.21 | 1.78 | -1.24 | S81478_s_at | protein amino acid dephosphorylation |
| **E** | Cxcr4 | 1.44 | 2.82 | 1.96 | U90610_at | response to hypoxia |
| **E** | Hpcal1 | 1.36 | 3.15 | 2.31 | D13126_at | n/a |
| **F** | Cartpt | ns | -1.67 | -1.58 | U10071_at | activation of MAPKK activity |
| **F** | Thy1 | ns | 1.21 | 1.22 | X02002_at | angiogenesis |
| **F** | Hspb1 | ns | -1.44 | -1.53 | rc_AA998683_g_at | anti-apoptosis |
| **F** | Bok | ns | -1.31 | -1.31 | AF027954_at | apoptosis |
| **F** | Hrk | ns | -1.39 | -1.32 | rc_AI102299_s_at | apoptosis |
| **F** | Grm7 | ns | -1.91 | -1.79 | D16817_at | behavioral fear response |
| **F** | Vim | ns | -1.3 | -1.39 | X62952_at | biological_process |
| **F** | Pde4b | ns | -1.84 | -1.91 | M25350_s_at | cAMP catabolic process |
| **F** | Cd47 | ns | -1.49 | -1.47 | AF017437_at | cell adhesion |
| **F** | Ddc | ns | -1.36 | -1.3 | M84648mRNA_s_at | cellular amino acid and derivative metabolic process |
| **F** | Ralb | ns | 1.34 | 1.48 | L19699_g_at | cytokinesis |
| **F** | Mc4r | ns | -2.43 | -2 | U67863_at | diet induced thermogenesis |
| **F** | Kctd13 | ns | 1.29 | 1.33 | rc_AA859990_s_at | DNA replication |
| **F** | Kat5 | ns | 1.21 | 1.26 | rc_AA800738_at | double-strand break repair |
| **F** | Sh3kbp1 | ns | -1.5 | -1.33 | U90261UTR#1_g_at | endocytosis |
| **F** | Gsr | ns | -1.26 | -1.28 | U73174_g_at | glutathione metabolic process |
| **F** | Gch1 | ns | 1.93 | 1.72 | M58364_at | GTP catabolic process |
| **F** | Ptprr | ns | -1.81 | -1.71 | D64050_at | in utero embryonic development |
| **F** | Slc24a2 | ns | -1.59 | -1.36 | AF021923_at | ion transport |
| **F** | Slc12a7 | ns | 1.49 | 1.29 | rc_AA799691_at | ion transport |
| **F** | Tcn2 | ns | 1.36 | 1.34 | rc_AA893702_s_at | ion transport |
| **F** | Scn7a | ns | 1.92 | 2.3 | rc_AA925248_at | ion transport |
| **F** | Htr3a | ns | 1.36 | 1.4 | U59672_at | ion transport |
| **F** | Kcnc1 | ns | 1.25 | 1.29 | X62840mRNA_s_at | ion transport |
| **F** | Scn3a | ns | -1.21 | -1.2 | Y00766_at | ion transport |
| **F** | Thra | ns | -1.22 | -1.21 | M31174_at | kidney development |
| **F** | Dcn | ns | -1.61 | -1.61 | Z12298cds_s_at | kidney development |
| **F** | Acsl1 | ns | -1.45 | -1.34 | D90109_at | lipid metabolic process |
| **F** | Schip1 | ns | -1.37 | -1.24 | rc_AA800036_at | luteinization |
| **F** | Mark1 | ns | 1.36 | 1.37 | rc_AA800063_at | microtubule cytoskeleton organization |
| **F** | Mapt | ns | -1.42 | -1.37 | rc_AI227608_s_at | microtubule cytoskeleton organization |
| **F** | Ina | ns | -1.38 | -1.21 | rc_AA875659_s_at | multicellular organismal development |
| **F** | Csrp2 | ns | -2.38 | -2.29 | U44948_at | multicellular organismal development |
| **F** | Ptprm | ns | 1.21 | 1.23 | rc_AI639001_at | negative regulation of endothelial cell proliferation |
| **F** | Map1a | ns | -1.3 | -1.21 | M83196_at | negative regulation of microtubule depolymerization |
| **F** | Rgs4 | ns | -2.6 | -2.38 | U27767_at | negative regulation of signal transduction |
| **F** | Egr1 | ns | 2.08 | 2.8 | M18416_at | negative regulation of transcription from RNA polymerase II promoter |
| **F** | Htr2b | ns | -2.2 | -1.92 | X66842_at | neural crest cell migration |
| **F** | Syn2 | ns | 1.49 | 1.47 | M27925_at | neurotransmitter secretion |
| **F** | Gas6 | ns | -1.47 | -1.5 | D42148_at | organ regeneration |
| **F** | Adcyap1 | ns | -2.06 | -1.81 | rc_AI228407_s_at | ovarian follicle development |
| **F** | Loxl1 | ns | -1.49 | -1.41 | rc_AA859805_at | oxidation reduction |
| **F** | Plcb1 | ns | -1.94 | -1.94 | L14323_at | oxygen and reactive oxygen species metabolic process |
| **F** | Cds1 | ns | -1.3 | -1.44 | AB009999_g_at | phospholipid biosynthetic process |
| **F** | Fntb | ns | 1.28 | 1.22 | rc_AI230914_at | positive regulation of cell proliferation |
| **F** | Fgfbp3 | ns | 1.49 | 1.44 | rc_AA800782_at | positive regulation of vascular permeability |
| **F** | Plcb3 | ns | 1.96 | 1.62 | M99567_at | post-Golgi vesicle-mediated transport |
| **F** | Ptgs2 | ns | -1.47 | -1.29 | S67722_s_at | prostaglandin biosynthetic process |
| **F** | Cask | ns | -1.49 | -1.34 | U47110_at | protein complex assembly |
| **F** | Pcsk2 | ns | -1.34 | -1.24 | M83746_at | proteolysis |
| **F** | Ctsl1 | ns | 1.27 | 1.24 | rc_AI176595_s_at | proteolysis |
| **F** | Ecel1 | ns | -1.25 | -1.31 | Y16188_at | proteolysis |
| **F** | Sdcbp | ns | -1.29 | -1.2 | rc_AA892373_at | Ras protein signal transduction |
| **F** | Igfbp3 | ns | -2.41 | -1.78 | M31837_at | regulation of cell growth |
| **F** | Eed | ns | 1.31 | 1.37 | rc_AA799481_at | regulation of gene expression by genetic imprinting |
| **F** | Sstr2 | ns | 1.56 | 1.28 | M93273_at | regulation of muscle contraction |
| **F** | Gria2 | ns | -1.29 | -1.31 | M36419_s_at | regulation of receptor recycling |
| **F** | Bhlhe41 | ns | -1.63 | -2.07 | AF009329_at | regulation of transcription, DNA-dependent |
| **F** | Jund | ns | -1.36 | -1.28 | D26307cds_at | regulation of transcription, DNA-dependent |
| **F** | Accn1 | ns | -1.37 | -1.32 | U53211_at | response to acid |
| **F** | Egln3 | ns | -2.26 | -2.02 | rc_AA799678_s_at | response to hypoxia |
| **F** | Camk2d | ns | -1.29 | -1.29 | rc_AA894330_s_at | response to hypoxia |
| **F** | Hsd11b2 | ns | 3.07 | 2.47 | U22424_at | response to hypoxia |
| **F** | Pdlim1 | ns | -1.47 | -1.29 | U23769_at | response to hypoxia |
| **F** | Maob | ns | -1.75 | -1.64 | M23601_at | response to toxin |
| **F** | Lphn2 | ns | -1.43 | -1.55 | AF063102_at | signal transduction |
| **F** | Grb10 | ns | -1.44 | -1.41 | rc_AA800686_at | signal transduction |
| **F** | Adora2a | ns | -1.3 | -1.38 | S47609_s_at | synaptic transmission, dopaminergic |
| **F** | Cga | ns | -1.73 | -1.69 | D00575_at | thyroid hormone generation |
| **F** | Gfra2 | ns | 3.3 | 2.74 | U97143_at | transmembrane receptor protein tyrosine kinase signaling pathway |
| **F** | Asl | ns | -1.3 | -1.37 | D13978_s_at | urea cycle |
| **F** | Calb1 | ns | -1.74 | -1.44 | M31178_g_at | ureteric bud development |
| **F** | Sdc1 | ns | -1.28 | -1.32 | S61865_s_at | ureteric bud development |
| **F** | Sctr | ns | 1.55 | 1.42 | E04128cds_s_at | n/a |
| **F** | Nap1l1 | ns | -1.25 | -1.22 | rc_AA859920_at | n/a |
| **F** | Twf1 | ns | 1.26 | 1.23 | rc_AA892851_g_at | n/a |
| **F** | Tlcd1 | ns | 1.39 | 1.47 | rc_AI639187_at | n/a |
| **F** | Tmeff1 | ns | 1.9 | 1.79 | rc_AI639427_at | n/a |
| **F** | Tmem47 | ns | 2.27 | 1.55 | rc_AI639501_s_at | n/a |
| **F** | Trib3 | ns | -1.48 | -1.47 | rc_H31287_g_at | n/a |
| **F** | Maoa | ns | -1.76 | -1.67 | S45812_s_at | n/a |
| **F** | Elavl4 | ns | 1.28 | 1.34 | S83320_g_at | n/a |

^a^Venn diagram illustrated in Figure 3; ^b^FC = fold change.
