# Supplementary material for: Transcriptional Responses of Cultured Rat Sympathetic Neurons during BMP-7-Induced Dendritic Growth
Source: PLoS One. 2011 Jul 13;6(7):e21754. doi: 10.1371/journal.pone.0021754 (PMC3135585; doi:10.1371/journal.pone.0021754)
Supplement: Table S5 — Genes significantly changed at BMP24h relative to BMP6h (156 total). aVenn diagram illustrated in Figure 3; bFC = fold change. (DOC) [file pone.0021754.s005.doc]

**Table S5. Genes significantly changed at BMP24h relative to BMP6h (156 total)**.

| **Venn Diagram Section^a^** | **Gene Symbol** | **FC^b^ BMP6h vs Control** | **FC BMP24h vs Control** | **FC BMP24h vs BMP6h** | **Probe Set ID** | **GO Biological Process** |
| --- | --- | --- | --- | --- | --- | --- |
| **D** | Hsd17b6 | -1.3 | ns | 1.35 | U89280_at | androgen metabolic process |
| **D** | Nr3c1 | -1.36 | ns | 1.35 | rc_AA893618_s_at | chromatin remodeling |
| **D** | Klf9 | 1.87 | ns | -1.71 | D12769_g_at | embryo implantation |
| **D** | Map3k1 | 1.56 | ns | -1.59 | U48596_g_at | MAPKKK cascade |
| **D** | Prrxl1 | 2 | ns | -2.01 | U29174_at | neuron migration |
| **D** | Rxrg | 1.7 | ns | -1.5 | AF016387_g_at | regulation of transcription, DNA-dependent |
| **D** | Imp3 | -1.4 | ns | 1.3 | rc_AA799369_at | rRNA processing |
| **D** | Ttr | -1.32 | ns | 1.31 | rc_AA945169_at | transport |
| **D** | Gse1 | 1.43 | ns | -1.24 | rc_AI013993_at | n/a |
| **D** | Pmepa1 | 1.86 | ns | -1.55 | rc_AI639058_s_at | n/a |
| **E** | Ngfr | 1.66 | 3.77 | 2.27 | X05137_at | apoptosis |
| **E** | Jag1 | 1.66 | 2.84 | 1.71 | L38483_at | cell fate determination |
| **E** | Olfm1 | 1.25 | 1.79 | 1.43 | U03414_s_at | multicellular organismal development |
| **E** | Id2 | 5.37 | 3.96 | -1.36 | rc_AI137583_at | negative regulation of transcription from RNA polymerase II promoter |
| **E** | Dusp1 | 2.21 | 1.78 | -1.24 | S81478_s_at | protein amino acid dephosphorylation |
| **E** | Cxcr4 | 1.44 | 2.82 | 1.96 | U90610_at | response to hypoxia |
| **E** | Hpcal1 | 1.36 | 3.15 | 2.31 | D13126_at | n/a |
| **F** | Cartpt | ns | -1.67 | -1.58 | U10071_at | activation of MAPKK activity |
| **F** | Thy1 | ns | 1.21 | 1.22 | X02002_at | angiogenesis |
| **F** | Hspb1 | ns | -1.44 | -1.53 | rc_AA998683_g_at | anti-apoptosis |
| **F** | Bok | ns | -1.31 | -1.31 | AF027954_at | apoptosis |
| **F** | Hrk | ns | -1.39 | -1.32 | rc_AI102299_s_at | apoptosis |
| **F** | Grm7 | ns | -1.91 | -1.79 | D16817_at | behavioral fear response |
| **F** | Vim | ns | -1.3 | -1.39 | X62952_at | biological_process |
| **F** | Pde4b | ns | -1.84 | -1.91 | M25350_s_at | cAMP catabolic process |
| **F** | Cd47 | ns | -1.49 | -1.47 | AF017437_at | cell adhesion |
| **F** | Ddc | ns | -1.36 | -1.3 | M84648mRNA_s_at | cellular amino acid and derivative metabolic process |
| **F** | Ralb | ns | 1.34 | 1.48 | L19699_g_at | cytokinesis |
| **F** | Mc4r | ns | -2.43 | -2 | U67863_at | diet induced thermogenesis |
| **F** | Kctd13 | ns | 1.29 | 1.33 | rc_AA859990_s_at | DNA replication |
| **F** | Kat5 | ns | 1.21 | 1.26 | rc_AA800738_at | double-strand break repair |
| **F** | Sh3kbp1 | ns | -1.5 | -1.33 | U90261UTR#1_g_at | endocytosis |
| **F** | Gsr | ns | -1.26 | -1.28 | U73174_g_at | glutathione metabolic process |
| **F** | Gch1 | ns | 1.93 | 1.72 | M58364_at | GTP catabolic process |
| **F** | Ptprr | ns | -1.81 | -1.71 | D64050_at | in utero embryonic development |
| **F** | Slc24a2 | ns | -1.59 | -1.36 | AF021923_at | ion transport |
| **F** | Slc12a7 | ns | 1.49 | 1.29 | rc_AA799691_at | ion transport |
| **F** | Tcn2 | ns | 1.36 | 1.34 | rc_AA893702_s_at | ion transport |
| **F** | Scn7a | ns | 1.92 | 2.3 | rc_AA925248_at | ion transport |
| **F** | Htr3a | ns | 1.36 | 1.4 | U59672_at | ion transport |
| **F** | Kcnc1 | ns | 1.25 | 1.29 | X62840mRNA_s_at | ion transport |
| **F** | Scn3a | ns | -1.21 | -1.2 | Y00766_at | ion transport |
| **F** | Thra | ns | -1.22 | -1.21 | M31174_at | kidney development |
| **F** | Dcn | ns | -1.61 | -1.61 | Z12298cds_s_at | kidney development |
| **F** | Acsl1 | ns | -1.45 | -1.34 | D90109_at | lipid metabolic process |
| **F** | Schip1 | ns | -1.37 | -1.24 | rc_AA800036_at | luteinization |
| **F** | Mark1 | ns | 1.36 | 1.37 | rc_AA800063_at | microtubule cytoskeleton organization |
| **F** | Mapt | ns | -1.42 | -1.37 | rc_AI227608_s_at | microtubule cytoskeleton organization |
| **F** | Ina | ns | -1.38 | -1.21 | rc_AA875659_s_at | multicellular organismal development |
| **F** | Csrp2 | ns | -2.38 | -2.29 | U44948_at | multicellular organismal development |
| **F** | Ptprm | ns | 1.21 | 1.23 | rc_AI639001_at | negative regulation of endothelial cell proliferation |
| **F** | Map1a | ns | -1.3 | -1.21 | M83196_at | negative regulation of microtubule depolymerization |
| **F** | Rgs4 | ns | -2.6 | -2.38 | U27767_at | negative regulation of signal transduction |
| **F** | Egr1 | ns | 2.08 | 2.8 | M18416_at | negative regulation of transcription from RNA polymerase II promoter |
| **F** | Htr2b | ns | -2.2 | -1.92 | X66842_at | neural crest cell migration |
| **F** | Syn2 | ns | 1.49 | 1.47 | M27925_at | neurotransmitter secretion |
| **F** | Gas6 | ns | -1.47 | -1.5 | D42148_at | organ regeneration |
| **F** | Adcyap1 | ns | -2.06 | -1.81 | rc_AI228407_s_at | ovarian follicle development |
| **F** | Loxl1 | ns | -1.49 | -1.41 | rc_AA859805_at | oxidation reduction |
| **F** | Plcb1 | ns | -1.94 | -1.94 | L14323_at | oxygen and reactive oxygen species metabolic process |
| **F** | Cds1 | ns | -1.3 | -1.44 | AB009999_g_at | phospholipid biosynthetic process |
| **F** | Fntb | ns | 1.28 | 1.22 | rc_AI230914_at | positive regulation of cell proliferation |
| **F** | Fgfbp3 | ns | 1.49 | 1.44 | rc_AA800782_at | positive regulation of vascular permeability |
| **F** | Plcb3 | ns | 1.96 | 1.62 | M99567_at | post-Golgi vesicle-mediated transport |
| **F** | Ptgs2 | ns | -1.47 | -1.29 | S67722_s_at | prostaglandin biosynthetic process |
| **F** | Cask | ns | -1.49 | -1.34 | U47110_at | protein complex assembly |
| **F** | Pcsk2 | ns | -1.34 | -1.24 | M83746_at | proteolysis |
| **F** | Ctsl1 | ns | 1.27 | 1.24 | rc_AI176595_s_at | proteolysis |
| **F** | Ecel1 | ns | -1.25 | -1.31 | Y16188_at | proteolysis |
| **F** | Sdcbp | ns | -1.29 | -1.2 | rc_AA892373_at | Ras protein signal transduction |
| **F** | Igfbp3 | ns | -2.41 | -1.78 | M31837_at | regulation of cell growth |
| **F** | Eed | ns | 1.31 | 1.37 | rc_AA799481_at | regulation of gene expression by genetic imprinting |
| **F** | Sstr2 | ns | 1.56 | 1.28 | M93273_at | regulation of muscle contraction |
| **F** | Gria2 | ns | -1.29 | -1.31 | M36419_s_at | regulation of receptor recycling |
| **F** | Bhlhe41 | ns | -1.63 | -2.07 | AF009329_at | regulation of transcription, DNA-dependent |
| **F** | Jund | ns | -1.36 | -1.28 | D26307cds_at | regulation of transcription, DNA-dependent |
| **F** | Accn1 | ns | -1.37 | -1.32 | U53211_at | response to acid |
| **F** | Egln3 | ns | -2.26 | -2.02 | rc_AA799678_s_at | response to hypoxia |
| **F** | Camk2d | ns | -1.29 | -1.29 | rc_AA894330_s_at | response to hypoxia |
| **F** | Hsd11b2 | ns | 3.07 | 2.47 | U22424_at | response to hypoxia |
| **F** | Pdlim1 | ns | -1.47 | -1.29 | U23769_at | response to hypoxia |
| **F** | Maob | ns | -1.75 | -1.64 | M23601_at | response to toxin |
| **F** | Lphn2 | ns | -1.43 | -1.55 | AF063102_at | signal transduction |
| **F** | Grb10 | ns | -1.44 | -1.41 | rc_AA800686_at | signal transduction |
| **F** | Adora2a | ns | -1.3 | -1.38 | S47609_s_at | synaptic transmission, dopaminergic |
| **F** | Cga | ns | -1.73 | -1.69 | D00575_at | thyroid hormone generation |
| **F** | Gfra2 | ns | 3.3 | 2.74 | U97143_at | transmembrane receptor protein tyrosine kinase signaling pathway |
| **F** | Asl | ns | -1.3 | -1.37 | D13978_s_at | urea cycle |
| **F** | Calb1 | ns | -1.74 | -1.44 | M31178_g_at | ureteric bud development |
| **F** | Sdc1 | ns | -1.28 | -1.32 | S61865_s_at | ureteric bud development |
| **F** | Sctr | ns | 1.55 | 1.42 | E04128cds_s_at | n/a |
| **F** | Nap1l1 | ns | -1.25 | -1.22 | rc_AA859920_at | n/a |
| **F** | Twf1 | ns | 1.26 | 1.23 | rc_AA892851_g_at | n/a |
| **F** | Tlcd1 | ns | 1.39 | 1.47 | rc_AI639187_at | n/a |
| **F** | Tmeff1 | ns | 1.9 | 1.79 | rc_AI639427_at | n/a |
| **F** | Tmem47 | ns | 2.27 | 1.55 | rc_AI639501_s_at | n/a |
| **F** | Trib3 | ns | -1.48 | -1.47 | rc_H31287_g_at | n/a |
| **F** | Maoa | ns | -1.76 | -1.67 | S45812_s_at | n/a |
| **F** | Elavl4 | ns | 1.28 | 1.34 | S83320_g_at | n/a |
| **G** | Capza2 | ns | ns | 1.25 | rc_AA892842_at | actin cytoskeleton organization |
| **G** | Atp1a1 | ns | ns | -1.29 | M74494_g_at | ATP catabolic process |
| **G** | Sfxn3 | ns | ns | 1.21 | rc_AA891880_at | cation transport |
| **G** | Rgc32 | ns | ns | -1.52 | AF036548_g_at | cell cycle |
| **G** | Mapk14 | ns | ns | -1.23 | U73142_g_at | DNA damage checkpoint |
| **G** | Pura | ns | ns | -1.32 | rc_AI229291_at | DNA unwinding involved in replication |
| **G** | Cd55 | ns | ns | -1.45 | AF039583_s_at | elevation of cytosolic calcium ion concentration |
| **G** | Gcgr | ns | ns | 2.05 | M96674_at | exocytosis |
| **G** | Sc4mol | ns | ns | 1.22 | rc_AI172293_at | fatty acid biosynthetic process |
| **G** | Gstm1 | ns | ns | -1.27 | H32189_s_at | glutathione metabolic process |
| **G** | Gsta3 | ns | ns | -1.27 | X78848cds_f_at | glutathione metabolic process |
| **G** | Rabep1 | ns | ns | 1.24 | D85844_at | Golgi to endosome transport |
| **G** | Alad | ns | ns | 1.39 | rc_AA800745_at | heme biosynthetic process |
| **G** | Clcn3 | ns | ns | -1.2 | D17521_at | ion transport |
| **G** | Acsl3 | ns | ns | 1.23 | D30666_at | lipid metabolic process |
| **G** | Acadm | ns | ns | -1.44 | J02791_at | liver development |
| **G** | Pak1 | ns | ns | -1.42 | U49953_s_at | MAPKKK cascade |
| **G** | Smad1 | ns | ns | -1.31 | U66478_at | MAPKKK cascade |
| **G** | Aph1a | ns | ns | 1.21 | rc_AA800566_g_at | metanephros development |
| **G** | Nefl | ns | ns | -1.2 | M25638_s_at | microtubule cytoskeleton organization |
| **G** | Ppil3 | ns | ns | 1.31 | rc_AA892680_at | mRNA processing |
| **G** | Ednrb | ns | ns | 1.48 | X57764_s_at | neural crest cell migration |
| **G** | Dlc1 | ns | ns | -1.34 | D31962UTR#1_at | neural tube closure |
| **G** | Car5b | ns | ns | 1.34 | rc_AA892953_s_at | one-carbon metabolic process |
| **G** | Slc16a1 | ns | ns | -1.3 | rc_AI145680_s_at | organic anion transport |
| **G** | Fhl2 | ns | ns | 1.34 | rc_AA891527_at | osteoblast differentiation |
| **G** | Chkb | ns | ns | 1.29 | AB006607_g_at | phosphatidylethanolamine biosynthetic process |
| **G** | Plcb4 | ns | ns | -1.27 | AF027571_s_at | phototransduction |
| **G** | Arhgef9 | ns | ns | 1.84 | rc_AI639196_at | postsynaptic membrane organization |
| **G** | Ptprs | ns | ns | -1.23 | L19933_s_at | protein amino acid dephosphorylation |
| **G** | Map4k1 | ns | ns | 1.49 | rc_AA891302_g_at | protein amino acid phosphorylation |
| **G** | Fkbp4 | ns | ns | -1.22 | rc_AI231547_at | protein folding |
| **G** | Akap12 | ns | ns | -1.34 | U23146cds_s_at | protein targeting |
| **G** | Cpd | ns | ns | -1.25 | U62897_at | proteolysis |
| **G** | Cnot2 | ns | ns | 1.25 | rc_AA800637_at | regulation of transcription |
| **G** | Taf1c | ns | ns | 1.3 | rc_AA892987_at | regulation of transcription |
| **G** | Pou3f1 | ns | ns | -1.58 | M72711_at | regulation of transcription, DNA-dependent |
| **G** | Plod2 | ns | ns | -1.21 | rc_AA892897_at | response to hypoxia |
| **G** | Pld2 | ns | ns | 1.32 | rc_AA998338_s_at | response to hypoxia |
| **G** | P2rx2 | ns | ns | 1.36 | U14414_at | response to hypoxia |
| **G** | Adh5 | ns | ns | 1.29 | rc_AA874874_at | retinoid metabolic process |
| **G** | Sec11a | ns | ns | 1.25 | L11319_at | signal peptide processing |
| **G** | Gnb3 | ns | ns | -1.2 | L29090cds_at | signal transduction |
| **G** | Gnpat | ns | ns | 1.27 | rc_AA799779_g_at | synapse assembly |
| **G** | Sptbn2 | ns | ns | -1.21 | AB001347_s_at | synaptic vesicle exocytosis |
| **G** | Ppp1r10 | ns | ns | 1.29 | AF040954_at | transcription |
| **G** | Rbp1 | ns | ns | -1.23 | M19257_at | transport |
| **G** | Pex5 | ns | ns | 1.29 | rc_AA892300_at | very long-chain fatty acid metabolic process |
| **G** | Fn1 | ns | ns | -1.33 | L00191cds#1_s_at | n/a |
| **G** | Trim8 | ns | ns | -1.21 | rc_AA875001_at | n/a |
| **G** | Ankib1 | ns | ns | 1.3 | rc_AA875143_at | n/a |
| **G** | Lama3 | ns | ns | -1.45 | rc_AA946108_at | n/a |
| **G** | Nudt6 | ns | ns | 1.46 | rc_AA997476_at | n/a |
| **G** | Bmp3 | ns | ns | -1.55 | S77492_i_at | n/a |
| **G** | App | ns | ns | -1.21 | X07648cds_g_at | n/a |
| **G** | Apbb1 | ns | ns | -1.2 | X60468mRNA_s_at | n/a |
| **G** | Amd1 | ns | ns | 1.24 | Z15123exon#5_s_at | n/a |

^a^Venn diagram illustrated in Figure 3; ^b^FC = fold change.
